# Loss of C3 and CD14 reduces region-specific neuroinflammation in a murine polytrauma model

**DOI:** 10.1007/s00011-026-02367-7

**Published:** 2026-09-24

**Authors:** Florian Olde Heuvel, Marica Pagliarini, Fan Sun, Ludmila Lupu, Zongren Zhao, Likun Cui, Jin Zhang, Rebecca Halbgebauer, Marco Mannes, Tobias Maria Boeckers, Bernd Knöll, Egil Lien, Tom Eirik Mollnes, Markus Huber-Lang, Francesco Roselli

**Affiliations:** 1 Department of Neurology, UIm University, Ulm, Germany; 2 Institute of Clinical and Experimental Trauma Immunology, Ulm University Medical Center, Ulm, Germany; 3 Institute of Anatomy and Cell Biology, Ulm University, Ulm, Germany; 4 Institute of Neurobiochemistry, Ulm University, Ulm, Germany; 5 Division of Infectious Diseases and Immunology, Department of Medicine, University of Massachusetts Chan Medical School, Worcester, MA USA; 6 Department of Immunology, Oslo University Hospital, Oslo, Norway; 7 Research Laboratory, Nordland Hospital Trust, Bodoe, Norway; 8 German Center for Neurodegenerative Diseases (DZNE), Ulm, Germany; 9 Institute of Clinical and Experimental Trauma Immunology, Center Center for Biomedical Research (ZBF), Ulm University Medical, Helmholtzstrasse 8/1, 89081 Ulm, Germany; 10 Center for Biomedical Research (ZBF), Ulm University, Helmholtzstrasse 8/1, 89081 Ulm, Germany

**Keywords:** Delirium, Microglia, Complement, TNF, Cortex, Striatum, Polytrauma

## Abstract

**Background:**

TBI-polytrauma (P-TBI) is associated with acute neurological deterioration, delirium, and poor prognosis. Systemic inflammatory mediators are thought to amplify the cerebral neuroimmune response, particularly microglial activation, with detrimental consequences.

**Methods:**

We investigated the roles of complement factor C3 and the TLR co-receptor CD14 in a murine polytrauma model combining mild TBI with femur fracture, blunt thorax trauma, and resuscitated haemorrhagic shock, using mice lacking C3, CD14, or both.

**Results:**

P-TBI induced a rapid, brain-wide increase in inflammatory cytokines with region-specific patterns. *TNF* and *CCL2* mRNA were upregulated in microglia in the cortex, hippocampus, and striatum, and this response was abolished in *C3*^*−/−*^*CD14*^*−/−*^ mice. Analysis of single knockouts showed that cytokine induction depended on CD14 in the cortex, C3 in the striatum, and both in the hippocampus. In contrast, neither C3 nor CD14 influenced cytokine induction at the cortical lesion site. Interestingly, neuronal *cFOS* responses showed an inverse regional C3/CD14 dependency compared with inflammatory cytokine responses, with a cortical C3 dependency and striatal CD14 dependency.

**Conclusion:**

Thus, C3 and CD14 are dispensable for the acute response at the injury site but differentially regulate microglial and neuronal activation in remote brain regions, representing potential targets to reduce P-TBI–associated encephalopathy.

**Supplementary Information:**

The online version contains supplementary material available at https://doi.org/10.1007/s00011-026-02367-7.

## Introduction

Polytrauma (PT), defined as the occurrence of injury in at least three body domains and several alterations in physiological parameters [1] is associated with multi-organ damage and high mortality [2]. The frequent involvement of the brain is mainly direct and focal, so that traumatic brain injury (TBI) is part of the initial PT constellation (PT including TBI, henceforth abbreviated as P-TBI). However, non-focal and rather related to secondary injury processes like altered level of consciousness, inattention and disorganised thinking as parts of the delirium diagnosis, and memory impairment are reported by more than half of P-TBI patients. These neurological consequences predict an overall worse long-term health status [3].

P-TBI is characterized by strong systemic immune activation initiated by the release of damage associated molecular patterns (DAMPs) which trigger signaling through a number of receptors, including Toll-like Receptors and their co-receptor CD14 [4]. TLR/CD14 signaling has been associated with secondary damage and organ dysfunction appearing in P-TBI patients and corresponding animal models [5, 6].

The pathophysiology and thrombo-inflammatory response following P-TBI is also driven by biologically active products derived from multiple protease-dependent cascades, including the coagulation and complement system [7]. The latter is initiated within minutes after polytrauma and peaks within a few hours [8] and constitutes a major source of systemic inflammatory mediators in P-TBI, such as C3a and C5a anaphylatoxins, which can trigger innate immunity processes in periphery as well as the brain level [9].

Thus, complement activation and TLR/CD14 signaling are key players in the systemic inflammatory response following P-TBI [10] and therefore are likely candidates to drive cerebral neuroinflammatory responses, in particular at the level of microglia (which is responsive to TLR signaling and complement factors), and their impact of the functionality of large-scale neuronal circuits.

In fact, neurological complications such as encephalopathy and delirium, are frequently detected upon systemic inflammation, such as sepsis [11] and major surgery [12]. Since the appearance of septic encephalopathy is linked to systemic complement activation [13] and associated on one side with the induction of reactive microglia [14] and on the other side with circuit dysfunction initiated by complement factors (C1q and C3 [15]).

We have therefore hypothesized that central neuroimmune activation and microglial reactivity may similarly take place in P-TBI and may correlate with alterations in neuronal alterations. We set out to establish the early neuroinflammatory profile in P-TBI and to mechanistically address the role of C3 and/or CD14 on the early neuroimmune response and of neuronal injury and activity markers across brain structures, exploiting mice genetically lacking the complement factor C3 factor and/or the TLR CD14 co-signaling molecule.

## Results

### Early cortical neuroinflammation in acute TBI/ polytrauma

**Fig. 1:**
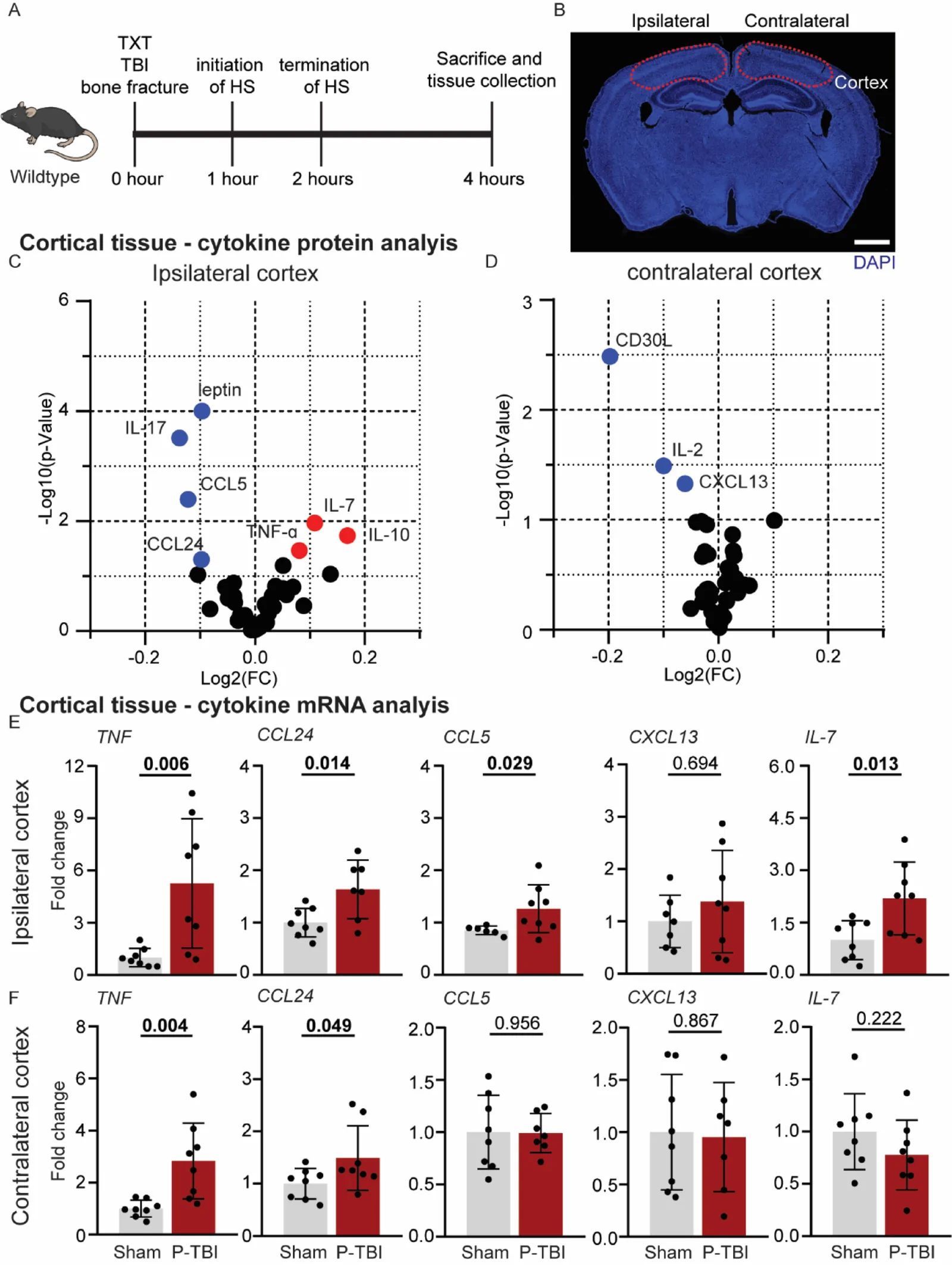
Polytrauma induces early cortical neuroinflammatory response. **A** Experimental design of the polytrauma, consisting of blunt thorax trauma (TXT), traumatic brain injury (TBI), bone fracture and haemorrhagic shock (HS). The total observation time post P-TBI is 4 h followed by sacrifice and tissue collection. **B** Representative image of DAPI stained in the mouse brain post P-TBI, highlighting the cortex. Scale bar: 200 μm. **C**,** D** Volcanoplot of cytokine protein analysis in cortical tissue revealed the significant upregulation of TNF-⍺, IL-7 and IL-10 and downregulation of CCL24, CCL5, IL-17 and leptin in the ipsilateral cortex. In the contralateral cortex significant downregulation of CXCL13, IL-2 and CD30L. **E**, **F** Cytokine mRNA analysis in ipsilateral cortical tissue revealed the significant upregulation of *TNF*, *CCL24*, *CCL5* and *IL-7* (*TNF*: *p* = 0.006, *CCL24*: *p* = 0.014, *CCL5*: *p* = 0.029, *IL-7*: *p* = 0.013). The contralateral cortex revealed a significant increase of *TNF* and *CCL24* (*TNF*: *p* = 0.004, *CCL24*: *p* = 0.05), with no difference in *CCL5*, *CXCL13* and *IL-7*. Data shown as volcanoplots depicting the Log2 fold change and -Log10* p*-value, and bar plots with individual data points. Group size: *N* = 6–8. Exact* p*-values are reported, significances are shown in bold

We set out to explore the acute neuroinflammatory responses occurring in the cerebral cortex upon TBI associated with Polytrauma (P-TBI), in particular in a mouse model involving mild TBI, thorax trauma, bone fracture and haemorrhagic shock (HS) or sham (Fig. 1A). We focused on the 4 h timepoint, compatible with timing for ICU admission of polytrauma patients [16]; at this stage, the overall architecture of the brain was preserved, and no macroscopic necrotic/haemorrhagic lesion was observed in any of sample (Fig. 1B).

First, we assessed the cytokine landscape in P-TBI (vs. shams) cortical samples either from the site of direct trauma (ipsilateral) or from the contralateral site using an antibody array covering 40 distinct inflammation mediators (Fig. 1C, D). The ipsilateral cortex displayed the significant elevation of TNF, IL-7 and IL-10, and the downregulation of CCL5 and CCL24 (Eotaxin-2; Fig. 1C). IL-17 and Leptin were also significantly downregulated, but their absolute intensities were very close to the detection limit. In the contralateral cortex the downregulation of CXCL13 and CD30L was detected (Fig. 1D; IL-2 was downregulated but at very low absolute levels).

Second, we verified the expression of a panel of mRNA of immune mediators related to the cytokine array findings. We detected the substantial upregulation of *TNF* mRNA both in ipsilateral (up to 12-fold the baseline; *p* = 0.006; Fig. 1E) and contralateral cortex (up to 5-fold; *p* = 0.004; Fig. 1F). *IL-7* mRNA was upregulated in the ipsilateral cortex only (*p* = 0.013; Fig. 1E, F). On the other hand, we detected the upregulation of the mRNA of *CCL24*/*Eotaxin-2* (ipsi: *p* = 0.014, contra: *p* = 0.049) and *CCL5* (*CCL5* ipsi: *p* = 0.029; Fig. 1E, F), opposite to the protein dynamics. No change in mRNA levels of *CXCL13* were detected in ipsi- or contralateral cortical samples (Fig. 1E, F).

Thus, P-TBI results in early and bilateral (ipsilateral/contralateral) neuroimmune activation as highlighted by the upregulation of TNF mRNA and protein level.

### Focal and diffuse neuroinflammation in acute TBI with polytrauma

**Fig. 2:**
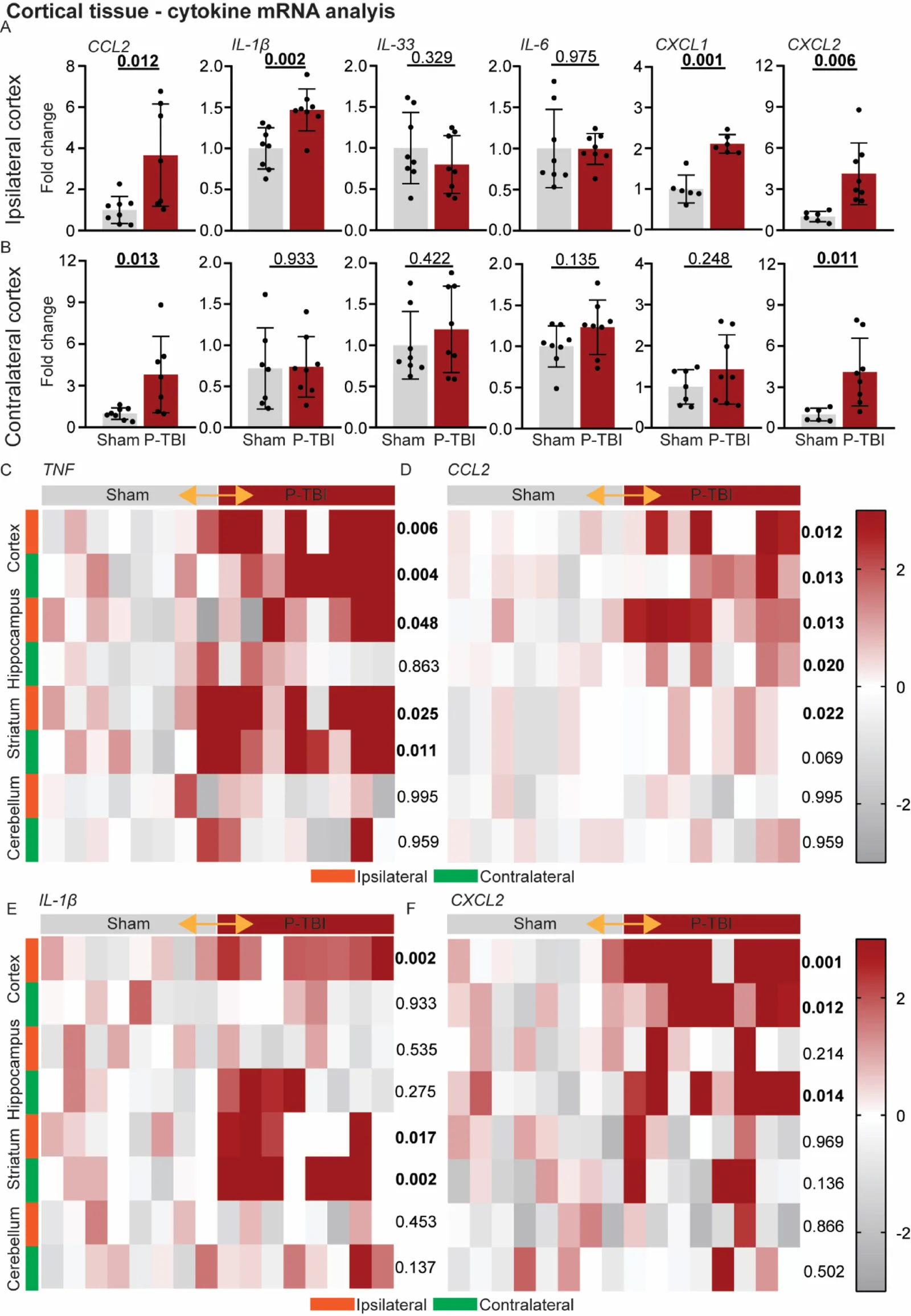
Focal and diffuse neuroinflammation in acute polytrauma with haemorrhagic shock. **A**, **B** Cytokine mRNA analysis in ipsilateral cortical tissue revealed the significant increase of *CCL2*, *IL-1β*,* CXCL1* and *CXCL2* (*CCL2*: *p* = 0.012, *IL-1β*: *p* = 0.002, *CXCL1*: *p* < 0.001, *CXCL2*: *p* = 0.006). Contralateral cortical tissue revealed a significant increase of *CCL2* and *CXCL2* (*CCL2*: *p* = 0.013, *CXCL2*: *p* = 0.011). No significant difference in *IL-33* and *IL-6* was observed. **C-F** Heatmaps depicting *TNF*, *CCL2*, *IL-1β* and *CXCL2* expression in both sham vs. P-TBI groups (comparison was made between sham and P-TBI; orange arrow, significance shown in stars next to the region) in ipsi- and contralateral cortex, hippocampus, striatum and cerebellum revealed large brain-wide increase of *TNF* (ipsi cortex: *p* = 0.006, contra cortex: *p* = 0.004, ipsi hippocampus: *p* = 0.048, ipsi striatum: *p* = 0.025, contra striatum: *p* = 0.011) and *CCL2* (ipsi cortex: *p* = 0.012, contra cortex: *p* = 0.013, ipsi hippocampus: *p* = 0.013, contra hippocampus: *p* = 0.02, ipsi striatum: *p* = 0.022 contra striatum: *p* = 0.069), in all regions except cerebellum. The upregulation of *IL-1β* was restricted to the ipsilateral cortex (*p* = 0.002) and both striata (Ipsi: *p* = 0.017; contra: *p* = 0.002) and the upregulation of *CXCL2* in both cortici (Ipsi: *p* = 0.001; contra: *p* = 0.012) and contralateral hippocampus (*p* = 0.014). Data shown as bar plots with individual data points and heatmaps depicting z-scores. Group size: *N* = 6–8. Exact* p*-values are reported, significances are shown in bold

The rapid and substantial elevation in *TNF* in both ipsi- and contralateral cortical samples suggested the early induction of innate-immunity mediators. We confirmed this view by assessing the expression of a set of cytokines with established early/innate response roles, namely *CCL2*,* IL-1β*,* IL-33*,* IL-6* and the murine IL-8 homologues *CXCL1* and *CXCL2*. *CCL2* mRNA was elevated both in ipsi- (*p* = 0.012; Fig. 2A) and in contralateral (*p* = 0.013; Fig. 2B) cortex, whereas *IL-1β* mRNA was upregulated only in the ipsilateral one (*p* = 0.002). Likewise, *CXCL2* was upregulated in both ipsi- (*p* = 0.006) and contra cortex (*p* = 0.011) whereas *CXCL1* was upregulated only in the ipsilateral one (*p* < 0.001; Fig. 2A-B). No elevation was detected for IL-33 and IL-6.

We exploited the upregulation of the mRNA of *TNF*, *CCL2*, *CXCL2* and *IL-1β* upon P-TBI to determine how P-TBI affected multiple brain structures, namely cortex, hippocampus, striatum and cerebellum, either ipsi- or contralateral. *TNF* and *CCL2* displayed a similar and widespread pattern of induction: substantial elevation of *TNF* and *CCL2* was detected in ipsi- and contralateral cortex (*TNF*: *p* = 0.006; *p* = 0.004; *CCL2*: *p* = 0.012; *p* = 0.013), in ipsilateral and contralateral striatum (*TNF*: *p* = 0.025; *p* = 0.011; *CCL2*: *p* = 0.022; *p* = 0.069) and hippocampus (*TNF*: *p* = 0.048; for contra, *p* = 0.863; *CCL*: *p* = 0.013; *p* = 0.02), though not in cerebellum (Fig. 2C-D). Conversely, *IL-1β* and *CXCL2* displayed a more restricted pattern of upregulation, both in ipsilateral (*IL-1β*: *p* = 0.002; *CXCL2*: *p* = 0.001) but only *CXCL2* also in contralateral cortex (*p* = 0.012) and hippocampus (*p* = 0.014) and only *IL-1β* in striatum (bilaterally; *p* = 0.017; *p* = 0.002; Fig. 2E-F). Again, no induction was noted in the cerebellum.

Thus, in the early P-TBI response, a structure-specific and site-specific inflammatory milieu may develop, with *TNF* and *CCL2* upregulated throughout cortex, hippocampus and striatum (but not cerebellum) and *IL-1β* overlapping with them only in ipsilateral cortex and striatum and *CXCL2* overlapping only in cortex.

Taken together, these findings highlight the co-occurrence of a focal, intense innate immunity activation, in correspondence of the ipsilateral cortex and a widespread, lower-intensity activation occurring across multiple ipsi- and contralateral brain structures.

### Loss of TNF, CCL2 induction and reactive microglia in mice lacking C3 and CD14

**Fig. 3:**
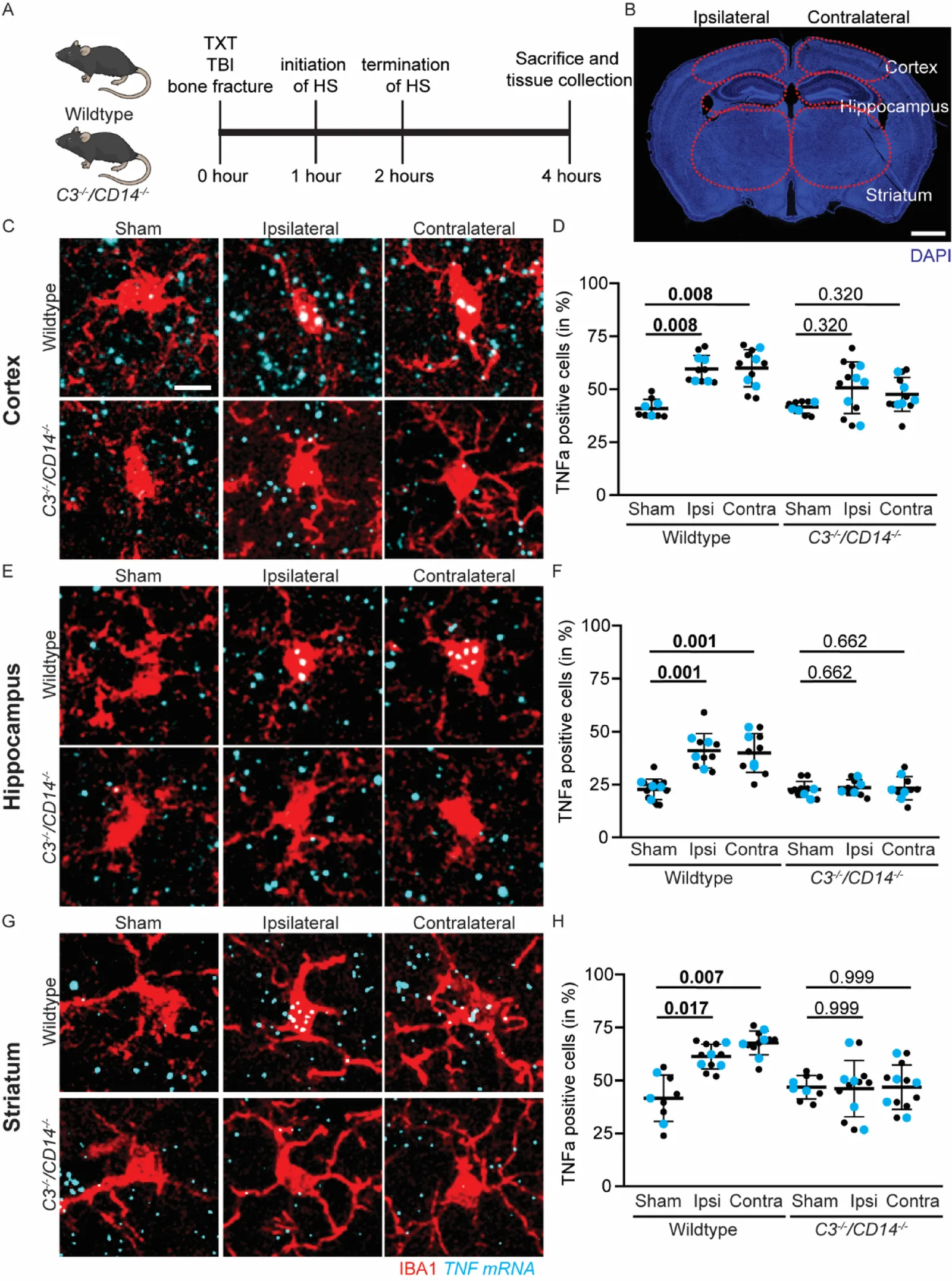
Brainwide Loss of P-TBI induced *TNF* expression in microglia of *C3*^*−/−*^*/CD14*^*−/−*^ mice. **A**, **B** Experimental design of polytrauma and TBI (P-TBI) in wildtype and *C3*^*−/−*^*/CD14*^*−/−*^ mice, investigating ipsilateral and contralateral cortex, hippocampus and striatum. **C–H** Single mRNA in situ hybridization of *TNF* in IBA1 + microglia revealed significant increase of *TNF+* microglia in ipsi- and contralateral cortex (WT sham vs. WT P-TBI ipsi: *p* = 0.008; WT sham vs. WT P-TBI contra: *p* = 0.008), hippocampus (WT sham vs. WT P-TBI ipsi: *p* = 0.0004; WT sham vs. WT P-TBI contra: *p* = 0.0004) and striatum (WT sham vs. WT P-TBI ipsi: *p* = 0.017; WT sham vs. WT P-TBI contra: *p* = 0.007; despite the non-significant omnibus analysis *p* = 0.135) of wildtype mice. No increase of *TNF+* microglia was observed in bilateral cortex, hippocampus or striatum for *C3*^*−/−*^*/CD14*^*−/−*^ mice. Data shown as dot plots with all data points depicted and single mice as coloured dots (cyan). Scale bar: 10 μm. Group size: *N* = 3, 4. Exact* p*-values are reported, significances are shown in bold

Complement factor 3 (C3) and CD14 have been recognized as critical mediator of the early systemic innate immunity response involved in secondary TBI [17, 18], in polytrauma [5] and in sepsis [19, 20], with their roles often redundant. We therefore explored the effect of the combined loss of C3 and CD14 on the early brain response to P-TBI. To this aim, we subjected either wild-type mice or *C3*^*−/−*^*CD14*^*−/−*^ double-knockout mice to P-TBI, obtained brain samples at the 4 h timepoint and subjected them to single-molecule hybridization for *TNF* or *CCL2* mRNA (Fig. 3A, B). We included the immunolabeling of microglia (using the IBA1 antigen), since we hypothesized that these cells may strongly contribute to the early inflammatory response and be sensitive to C3 and/or CD14 loss.

First, we quantified the overall density of *TNF* mRNA (number of molecules per surface unit, irrespective of their cellular source). Confirming the qPCR, mixed effect analysis revealed a significant increase between sham and P-TBI in all regions (Cortex: *p* = 0.006; hippocampus: *p* = 0.003; striatum: *p* = 0.0008). The prespecified pairwise comparison revealed a significant increase in cortex (ipsi: *p* = 0.004; contra: *p* = 0.004; Suppl. Fig. 1D, E), hippocampus (ipsi: *p* = 0.001; contra: *p* = 0.003; Suppl. Fig. 1, F) and striatum (ipsi: *p* = 0.004; contra: *p* = 0.004; Suppl. Fig. 1D, G) of WT mice. Specifically, microglial cells expressing *TNF* mRNA (containing at least 3 spots detected by in-situ hybridisation), were significantly increased upon P-TBI in cortex and hippocampus (Cortex: *p* = 0.007; hippocampus: *p* = 0.01; striatum: *p* = 0.135). The prespecified pairwise comparison revealed a significant increase in cortex (ipsi: *p* = 0.008; contra: *p* = 0.008; Fig. 3C, D), hippocampus (ipsi: *p* < 0.001; contra: *p* < 0.001; Fig. 1E, F) and striatum (ipsi: *p* = 0.017; contra: *p* = 0.007; Fig. 3G, H) of WT mice. These findings confirmed the upregulation of *TNF* mRNA and identified microglia as a prominent source.

Most notably, in *C3*^*−/−*^*CD14*^*−/−*^ mice, the increase in *TNF* mRNA was largely abrogated in the contralateral hippocampus (ipsi: *p* = 0.026; contra: *p* = 0.469; Suppl. Fig. 1D, F) and striatum (ipsi: *p* = 0.019; contra: *p* = 0.287; Suppl. Fig. 1D, G), but not in cortex (ipsi: *p* = 0.021; contra: *p* = 0.023; Suppl. Fig. 1D, E). However, focusing on *TNF* expression in microglia revealed the complete abrogation of induction in microglia in *C3*^*−/−*^*CD14*^*−/−*^ mice across cortex (ipsi: *p* = 0.32; contra: *p* = 0.32; Fig. C, D), hippocampus (ipsi: *p* = 0.662; contra: *p* = 0.662; Fig. 3E, F) and in the striatum (ipsi: *p* = 0.999; contra: *p* = 0.999; Fig. 3G, H). Of note, no differences in total microglia numbers were observed in any of the regions (Suppl. Fig. 2 A–C).

**Fig. 4:**
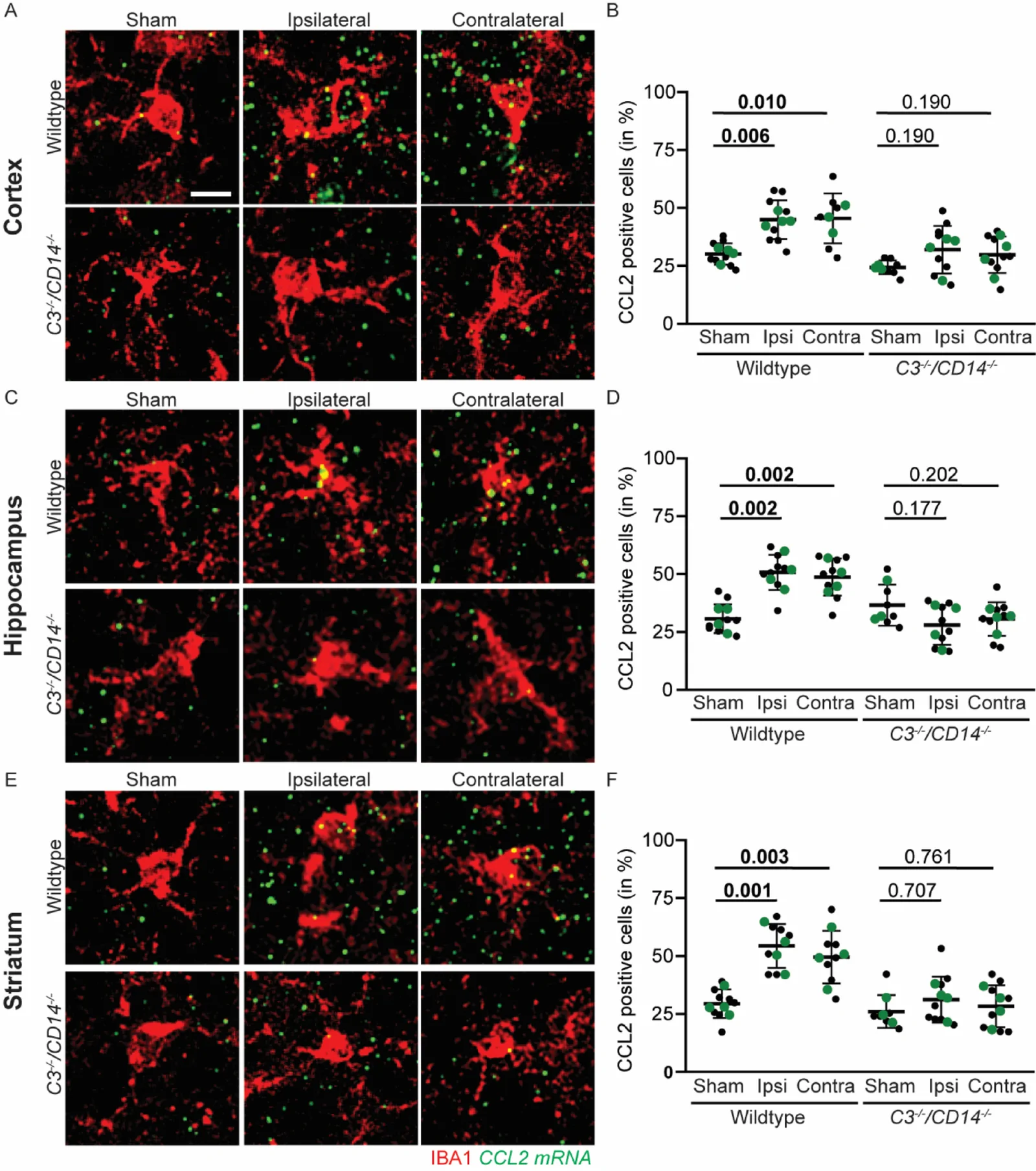
Brain wide Loss of P-TBI induced *CCL2* expression in microglia of *C3*^*−/−*^*/CD14*^*−/−*^ mice. **A–F** Single mRNA in situ hybridization of *CCL2* in IBA1 + microglia revealed significant increase of *CCL2 +* microglia in ipsi- and contralateral cortex (WT sham vs. WT P-TBI ipsi: *p* = 0.006; WT sham vs. WT P-TBI contra: *p* = 0.01), hippocampus (WT sham vs. WT P-TBI ipsi: *p* = 0.002; WT sham vs. WT P-TBI contra: *p* = 0.002, despite the non-significant omnibus analysis: *p* = 0.434) and striatum (WT sham vs. WT P-TBI ipsi: *p* = 0.001; WT sham vs. WT P-TBI contra: *p* = 0.003) of wildtype mice. No increase of *CCL2 +* microglia was observed in bilateral cortex, hippocampus or striatum for C3^−/−^/CD14^−/−^ mice. Data shown as dot plots with single sections depicted as black dots and single mice as coloured dots (green). Scale bar: 10 μm. Group size: *N* = 3, 4. Exact* p*-values are reported, significances are shown in bold

Second, we confirmed the *TNF* data by assessing the quantity and location of the *CCL2* mRNA. We found a substantial expression of *CCL2* in microglia, mixed effect analysis showed a significant increase in *CCL2* + microglia in P-TBI in cortex and striatum (Cortex: *p* = 0.042; hippocampus: *p* = 0.434; striatum: *p* = 0.029). The prespecified pairwise comparison revealed a significant increase in cortex (ipsi: *p* = 0.006; contra: *p* = 0.01; Fig. 4A, B), hippocampus (ipsi: *p* = 0.002; contra: *p* = 0.002; Fig. 4C, D) and striatum (ipsi: *p* = 0.001; contra: *p* = 0.003; Fig. 4E, F) of WT mice. On the other hand, no such increase in IBA1 + cells expressing *CCL2* mRNA + was observed in *C3*^*−/−*^*CD14*^*−/−*^ across cortex (ipsi: *p* = 0.19; contra: *p* = 0.19; Fig. 4A, B), hippocampus (ipsi: *p* = 0.177; contra: *p* = 0.202; Fig. 4C, D) nor striatum (ipsi: *p* = 0.707; contra: *p* = 0.761; Fig. 4E, F).

**Fig. 5:**
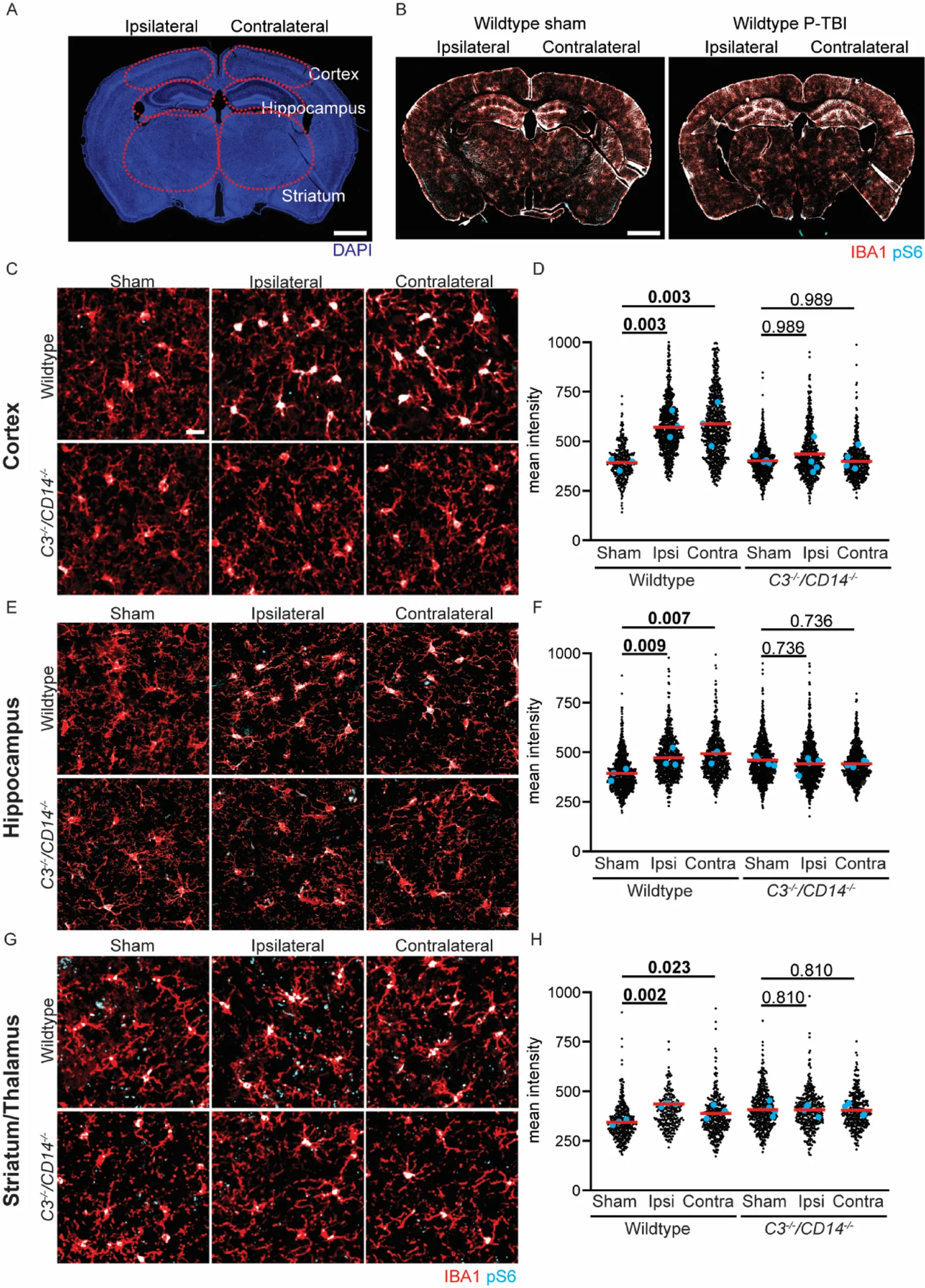
Loss of P-TBI induced microglia activity in *C3*^*−/−*^*/CD14*^*−/−*^ mice. **A, B** Representation of analyzed brain regions of wildtype mice post sham and P-TBI, depicting a brainwide induction of pS6 in IBA1 + microglia post P-TBI. **C–H** Immunofluorescence staining and imaging of pS6 and IBA1 revealed increase of pS6 intensity in microglia in both ipsi- and contralateral cortex (WT sham vs. WT P-TBI ipsi: *p* = 0.003; WT sham vs. WT P-TBI contra: *p* = 0.003), hippocampus (WT sham vs. WT P-TBI ipsi: *p* = 0.009; WT sham vs. WT P-TBI contra: *p* = 0.007; despite the non significant omnibus analysis: *p* = 0.108) and striatum (WT sham vs. WT P-TBI ipsi: *p* = 0.002; WT sham vs. WT P-TBI contra: *p* = 0.023). No increase of pS6 intensity was observed in bilateral cortex, hippocampus or striatum for *C3*^*−/−*^*/CD14*^*−/−*^ mice. Data shown as dot plots with single microglia depicted as black dots and single mice as coloured dots (cyan). Scale bar overview: 200 μm; insert: 10 μm. Group size: *N* = 3–4. Exact* p*-values are reported, significances are shown in bold

Third, we sought to confirm the effect of *C3*^*−/−*^*CD14*^*−/−*^ on the reactivity of microglia to P-TBI across the brain, using the phosphorylation of the ribosomal protein S6 as marker of cellular response. When phosphorylated, S6 enhances protein synthesis and S6 phosphorylation is used to identify cells undergoing active protein synthesis in response to stimuli [21, 22]. Immunolabelling intensity for pS6 in IBA1 + cells substantially increased in WT animals subject to P-TBI, mixed effect analysis revealed a significant increase in P-TBI in cortex and striatum, with a trend in hippocampus (Cortex: *p* = 0.035; hippocampus: *p* = 0.108; striatum: *p* = 0.013). The prespecified pairwise comparison revealed a significant increase in cortex (ipsi: *p* = 0.003; contra: *p* = 0.003; Fig. 5C, D), hippocampus (ipsi: *p* = 0.009; contra: *p* = 0.007; Fig. 5E, F) and striatum (ipsi: *p* = 0.002; contra: *p* = 0.023; Fig. 5G, H) of WT mice. Notably, the pairwise comparison revealed that also S6 phosphorylation induced by P-TBI in microglia was completely abolished in *C3*^*−/−*^*CD14*^*−/−*^ mice (Fig. 5C–H).

**Fig. 6:**
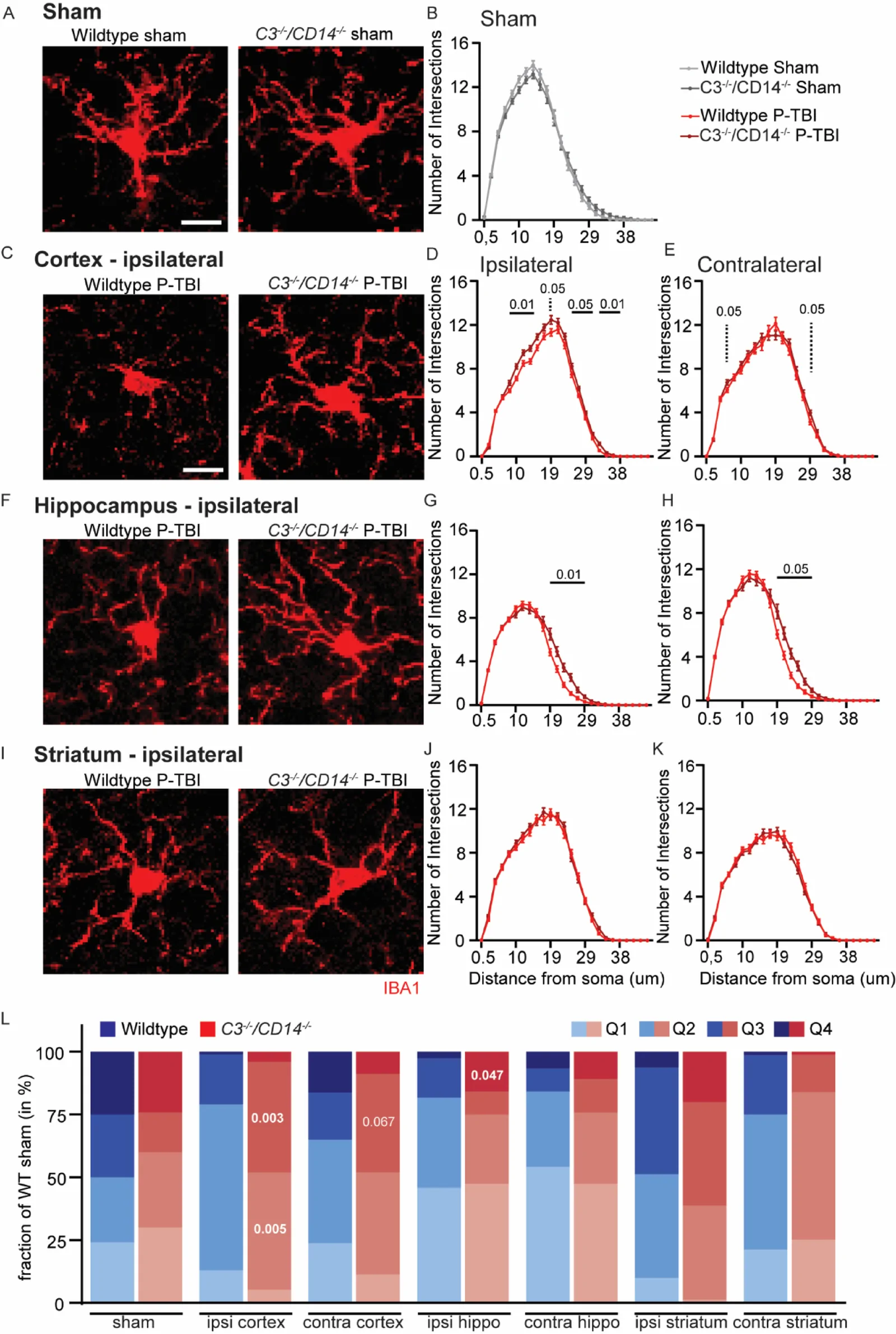
Loss of P-TBI induced reactive microglia morphology in C3^*−/−*^*/CD14*^*−/−*^ mice. **A, B** Immunofluorescence staining and imaging of IBA1 + microglia morphology revealed no differences between WT and *C3*^*−/−*^*/CD14*^*−/−*^ sham. **C–K** Immunofluorescence staining and imaging of IBA1 + microglia morphology revealed a decrease in the number of intersections between wildtype and *C3*^*−/−*^*/CD14*^*−/−*^ mice post P-TBI in ipsilateral cortex (WT P-TBI ipsi vs. *C3*^*−/−*^*/CD14*^*−/−*^ P-TBI ipsi: *p* < 0.05 at 8–16, 19, 25–27 and 32–36 μm) as well as ipsi- and contralateral hippocampus (WT P-TBI ipsi vs. *C3*^*−/−*^*/CD14*^*−/−*^ P-TBI ipsi: *p* < 0.05 at 19–30 μm; WT P-TBI contra vs. *C3*^*−/−*^*/CD14*^*−/−*^ P-TBI contra: *p* < 0.05 at 19–25 and 29 μm). No difference was observed in striatum. **L** Interquartile analysis, compared to WT sham, of all regions reveals that the *C3*^*−/−*^*/CD14*^*−/−*^ groups revealed a significant larger Q3 (*p* = 0.003) and significant smaller Q2 (*p* = 0.005) in the ipsilateral cortex and a strong trend towards increase of Q3 of contralateral cortex (*p* = 0.067) and significant increase in ipsilateral hippocampus (*p* = 0.047). Data shown as X-Y plots depicting number of intersections over distance from soma or percentage (per animal) of fractions of the WT sham group. Scale bar overview: 200 μm; 10 μm. Group size: *n* = 80–120 microglia per treatment group; *N* = 3–4 mice. Exact* p*-values are reported, significances are shown in bold

Finally, we examined the morphology of IBA1 + microglia, since reactive microglia is recognized to lose its ramified morphology in favour of an ameboid appearance [23]. Whereas microglia from WT and *C3*^*−/−*^*CD14*^*−/−*^ displayed a similar ramified architecture in sham animals (as quantified in the Sholl analysis; Fig. 6A, B), upon P-TBI, microglia in WT animals displayed a significant decrease in ramification compared to *C3*^*−/−*^*CD14*^*−/−*^ (thus, shifting toward an ameboid morphology) in the cortex (ipsi P-TBI: *p* < 0.05 at 8–16, 19, 25–27 and 32–36 μm; contra P-TBI: *p* < 0.05 at 6 and 29 μm; Fig. 6C–E) and hippocampus (ipsi P-TBI: *p* < 0.05 at 19–30 μm; contra P-TBI: *p* < 0.05 at 19–25 and 29 μm; Fig. 6F–H), though no change was observed in the striatum (Fig. 6I–K). Additionally, we performed an interquartile analysis of the total amount of intersections, compared to quartiles defined by the WT sham group. Interestingly an overall trend in increase of fractions of the two top quartiles (Q3 and Q4) and trend in decrease of fractions of the two lowest quartiles (Q1 and Q2) was observed between WT and *C3*^*−/−*^*CD14*^*−/−*^ microglia in each region. A significant increase was found in the ipsilateral cortex Q3 (*p* = 0.003), ipsilateral hippocampus Q4 (*p* = 0.047) and a strong trend in the contralateral cortex Q3 (*p* = 0.067). A significant decrease was observed in the ipsilateral cortex Q2 (*p* = 0.005).

Taken together, these findings show that microglia across cortex, hippocampus and striatum react to P-TBI with an increased transcription of *TNF* and *CCL2* mRNA, an upregulation of protein synthesis and a shift toward an ameboid morphology. Of note, all these manifestations of the reactive phenotype are abolished in animals lacking C3 and CD14.

### Induction of TNF and CCL2 upon P-TBI depends on C3 in cortex and striatum

**Fig. 7:**
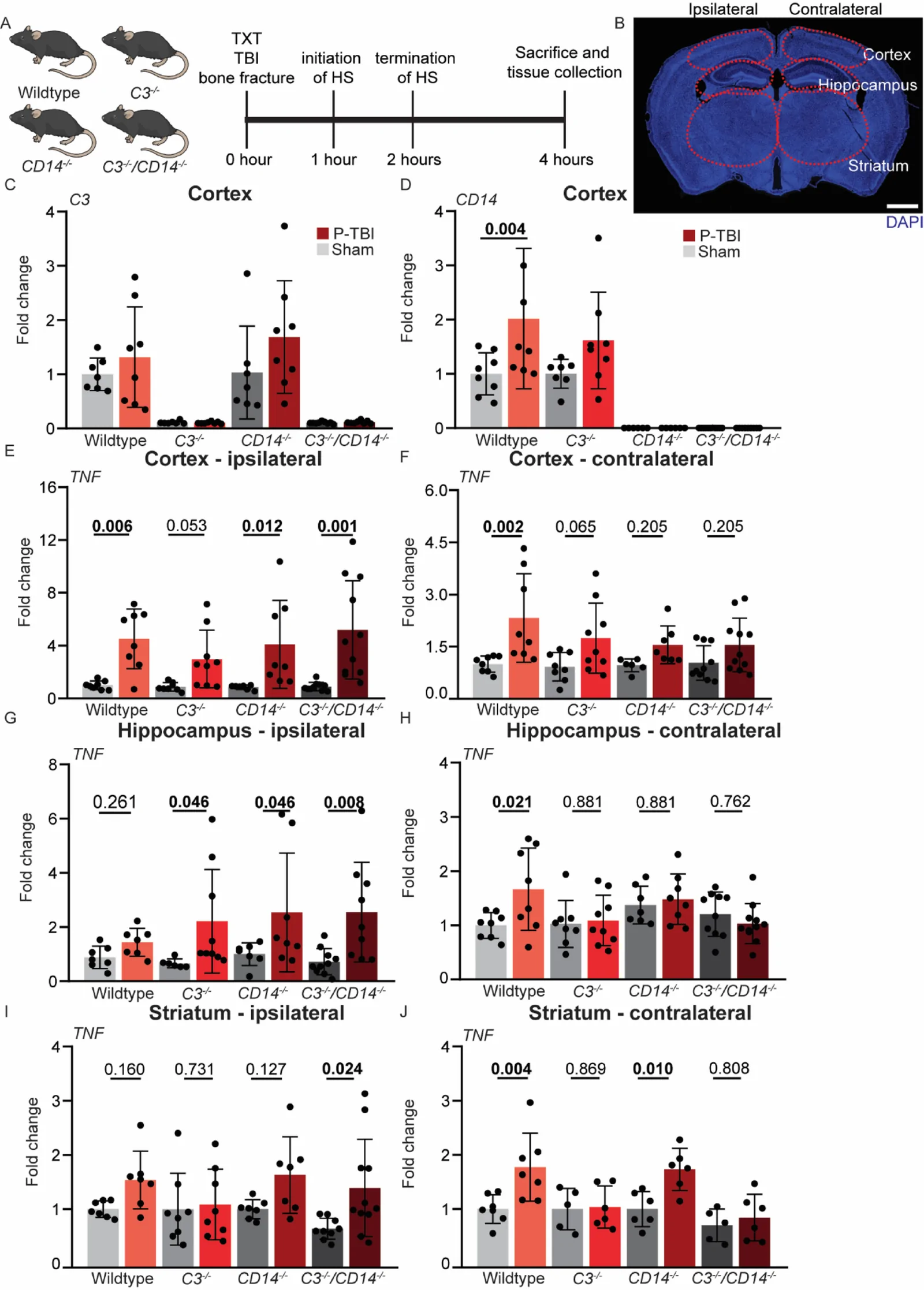
P-TBI induced *TNF* expression depends on CD14 in cortex and C3 in striatum. **A, B** Experimental design of polytrauma and TBI (P-TBI) in wildtype, *C3*^*−/−*^, *CD14*^*−/−*^ and *C3*^*−/−*^*/CD14*^*−/−*^ mice, investigating bilateral cortex, hippocampus and striatum. **C, D** Real-time quantitative PCR (RT-PCR) of *C3* and *CD14* expression in cortical tissue validates wildtype, *C3*^*−/−*^, *CD14*^*−/−*^ and *C3*^*−/−*^*/CD14*^*−/−*^ mice. **E, F** RT-PCR analysis of *TNF* expression in ipsi- and contralateral cortex post sham or P-TBI revealed an increase in all genotypes in the ipsilateral cortex (Sham vs. P-TBI; WT: *p* = 0.006; *C3*^*−/−*^: *p* = 0.053; *CD14*^*−/−*^: *p* = 0.012; *C3*^*−/−*^*/CD14*^*−/−*^: *p* = 0.001), with no differences in the P-TBI between the genotypes. In the contralateral cortex a significant increase was observed only in WT and a strong trend in *C3*^*−/−*^ mice (Sham vs. P-TBI; WT: *p* = 0.002; *C3*^*−/−*^: *p* = 0.065; *CD14*^*−/−*^: *p* = 0.205; *C3*^*−/−*^*/CD14*^*−/−*^: *p* = 0.205), with a trend to reduced TNF expression in the genotypes compared to WT P-TBI (*C3*^*−/−*^: *p* = 0.1; *CD14*^*−/−*^:: *p* = 0.09; *C3*^*−/−*^*/CD14*^*−/−*^: *p* = 0.07). **G, H** RT-PCR analysis of *TNF* expression in ipsi- and contralateral hippocampus post sham or P-TBI revealed a significant increase in all genotypes, with a trend in WT in the ipsilateral hippocampus (Sham vs. P-TBI; WT: *p* = 0.26; *C3*^*−/−*^: *p* = 0.046; *CD14*^*−/−*^: *p* = 0.046; *C3*^*−/−*^*/CD14*^*−/−*^: *p* = 0.008), with no differences in the P-TBI between the genotypes. In the contralateral hippocampus a significant increase was observed only in the WT (Sham vs. P-TBI; WT: *p* = 0.021; despite the non-significant omnibus analysis: *p* = 0.135), with a significant reduction in *TNF* expression in *C3*^*−/−*^ and *C3*^*−/−*^*/CD14*^*−/−*^ compared to WT (*C3*^*−/−*^: *p* = 0.023; *C3*^*−/−*^*/CD14*^*−/−*^: *p* = 0.012). **I-J.** RT-PCR analysis of *TNF* expression in ipsi- and contralateral striatum post sham and P-TBI revealed a significant increase in *C3*^*−/−*^*/CD14*^*−/−*^ with a trend in WT and *CD14*^*−/−*^ in the ipsilateral striatum (Sham vs. P-TBI; WT: *p* = 0.16; *C3*^*−/−*^: *p* = 0.731; *CD14*^*−/−*^: *p* = 0.13; *C3*^*−/−*^*/CD14*^*−/−*^: *p* = 0.024). In the contralateral striatum a significant increase was observed only in WT and *CD14*^*−/−*^ (Sham vs. P-TBI; wildtype: *p* = 0.004; CD14^−/−^: *p* = 0.01), with a significant reduction in *TNF* expression in *C3*^*−/−*^ and *C3*^*−/−*^*/CD14*^*−/−*^ compared to WT (*C3*^*−/−*^: *p* = 0.005; *C3*^*−/−*^*/CD14*^*−/−*^: *p* = 0.001). Data shown as bar plots with individual data points. Group size: *N* = 6–11. Exact* p*-values are reported, significances are shown in bold

Although C3 and CD14 usually reveal a converging role in driving inflammatory responses [10, 24], several instances are known in which their effects diverge or turn anti-inflammatory (e.g., CD14 is anti-inflammatory in the context of efferocytosis [25] whereas the C3 fragment iC3b suppresses proinflammatory cytokines from macrophages [26]). Since the double *C3*^*−/−*^*CD14*^*−/−*^ knock-out did not allow the disentanglement of any differential effect of C3 vs. CD14 in the early neuroimmune activation caused by P-TBI, we considered an in-depth investigation involving four distinct mouse genotypes: WT, single-KO *C3*^*−/−*^, single-KO *CD14*^*−/−*^ and double-KO *C3*^*−/−*^*CD14*^*−/−*^, each group divided in subgroups subjected to P-TBI or sham (altogether, 8 treatment x genotype groups; Fig. 7A). At the beginning, we verified that C3 mRNA was undetectable in *C3*^*−/−*^ and *C3*^*−/−*^*CD14*^*−/−*^ animals whereas CD14 mRNA was undetectable in *CD14*^*−/−*^ and *C3*^*−/−*^*CD14*^*−/−*^ animals (Fig. 7C, D).

First, we considered the induction of *TNF* mRNA (Fig. 3C, D) upon P-TBI across the four genotypes vs. sham controls for each genotype, examining ipsi and contralateral samples from cortex, hippocampus and striatum. Mixed effects analysis revealed a significant effect in sham vs. P-TBI in all regions, except contralateral hippocampus (ipsilateral cortex: *p* < 0.001; contralateral cortex: *p* = 0.0005; ipsilateral hippocampus: *p* = 0.002; contralateral hippocampus: *p* = 0.135; ipsilateral striatum: *p* = 0.005; contralateral striatum: *p* = 0.012). The pairwise comparison showed that for the ipsilateral cortex, *TNF* was upregulation in all genotypes (WT: *p* = 0.006; *C3*^*−/−*^: *p* = 0.053; *CD14*^*−/−*^: *p* = 0.012; *C3*^*−/−*^*CD14*^*−/−*^: *p* < 0.001; Fig. 7E), for the contralateral cortex however, an upregulation of *TNF* was observed only in WT and, to a lesser extent, *C3*^*−/−*^ (WT: *p* = 0.002; *C3*^*−/−*^: *p* = 0.065; Fig. 7F). In the ipsilateral hippocampus, the pairwise comparison revealed an upregulation in all genotypes, though with lesser extent in WT (WT: *p* = 0.261; *C3*^*−/−*^: *p* = 0.046; *CD14*^*−/−*^:: *p* = 0.046; *C3*^*−/−*^*CD14*^*−/−*^: *p* = 0.008; Fig. 7G), in the contralateral hippocampus, the upregulation of *TNF* was solely restricted to the WT animals (WT: *p* = 0.021; Fig. 7H). For the ipsilateral striatum, the upregulation of *TNF* was observed in the combined *C3*^*−/−*^*CD14*^*−/−*^, and to lesser extent WT and *CD14*^*−/−*^ (WT: *p* = 0.16; *CD14*^*−/−*^: *p* = 0.13; *C3*^*−/−*^*CD14*^*−/−*^: *p* = 0.024; Fig. 7I), for the contralateral striatum, an upregulation of *TNF* was only found in WT and *CD14*^*−/−*^ animals (WT: *p* = 0.004; *CD14*^*−/−*^: *p* = 0.01; Fig. 7J).

Second, the additional group of comparisons (P-TBI across genotypes) verified the results of the initial results, with no significances observed in the ipsilateral side. For the contralateral side a trend to decrease was found in the cortex for *CD14*^*−/−*^ and *C3*^*−/−*^*CD14*^*−/−*^ (*CD14*^*−/−*^: *p* = 0.086; *C3*^*−/−*^*CD14*^*−/−*^: *p* = 0.073; Fig. 7F), for the striatum a significant decrease was found in *C3*^*−/−*^ and *C3*^*−/−*^*CD14*^*−/−*^ (*C3*^*−/−*^: *p* = 0.005; *C3*^*−/−*^*CD14*^*−/−*^: *p* = 0.001; Fig. 7J). For the contralateral hippocampus, significant decreases were observed in *C3*^*−/−*^ and *C3*^*−/−*^*CD14*^*−/−*^. (full statistical descriptions are reported in supplementary file descriptive statistics)

Thus, *TNF* mRNA induction in the contralateral cortex suggests more a CD14 (than C3) dependency, striatum reveals a dependency on C3 whereas hippocampal *TNF* induction shows sensitivity to both C3 and CD14. In the ipsilateral cortex and hippocampus, the induction of *TNF* mRNA is not dependent on either C3 or CD14, whereas it appears dependent on C3 in the ipsilateral striatum.

**Fig. 8:**
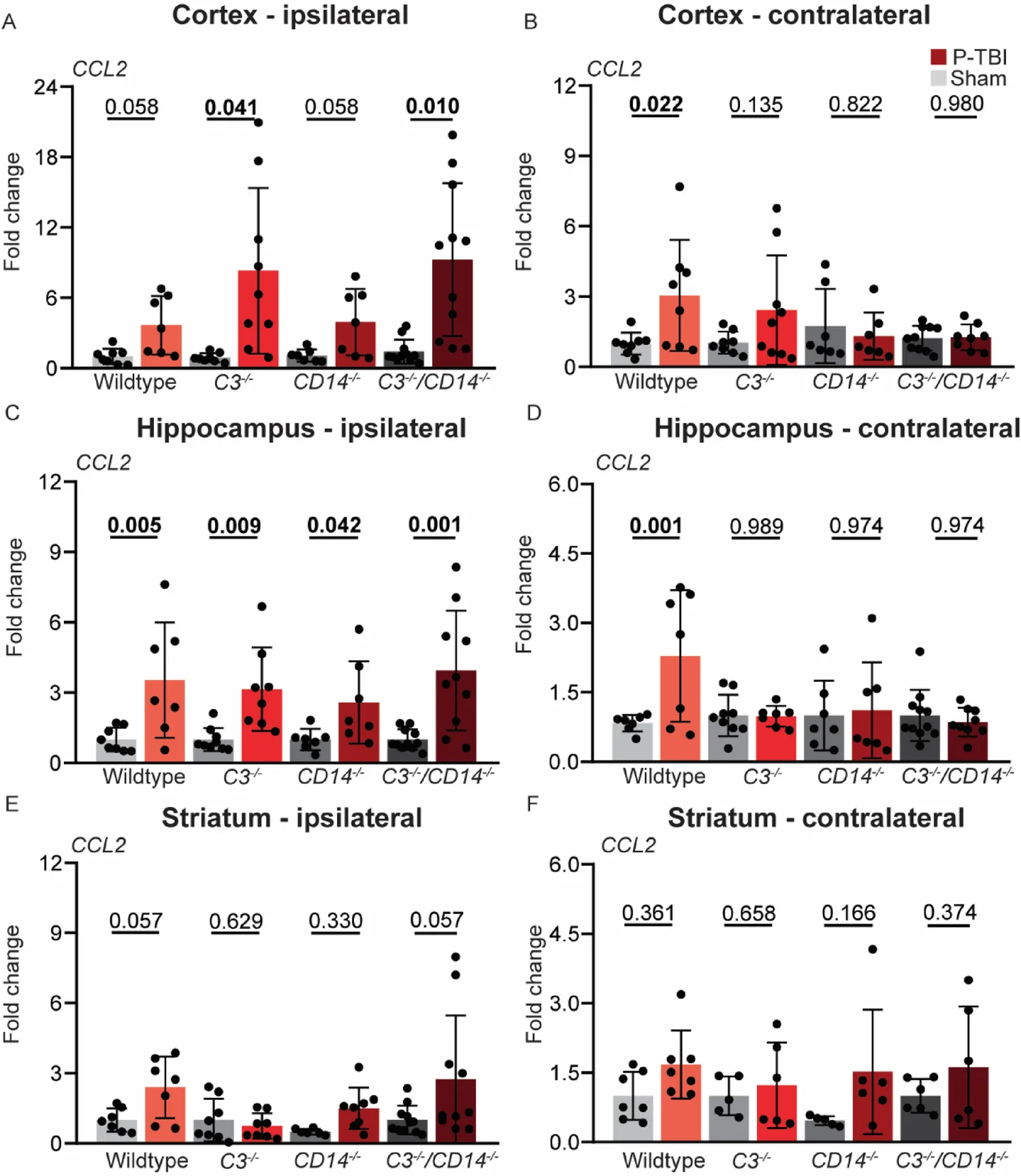
P-TBI induced *CCL2* expression in the cortex depends on CD14, and in the striatum depends on C3. **A, B** RT-PCR analysis of *CCL2* expression in ipsi- and contralateral cortex post sham and P-TBI revealed an increase in all genotypes in the ipsilateral cortex (Sham vs. P-TBI; WT: *p* = 0.059; *C3*^*−/−*^: *p* = 0.041; *CD14*^*−/−*^: *p* = 0.059; *C3*^*−/−*^*/CD14*^*−/−*^: *p* = 0.01), with a further increase of *CCL2* expression between *C3*^*−/−*^ and *C3*^*−/−*^*/CD14*^*−/−*^ compared with WT P-TBI (*C3*^*−/−*^: *p* = 0.045; *C3*^*−/−*^*/CD14*^*−/−*^: *p* = 0.014, despite the non-significant omnibus results: *p* = 0.084). In the contralateral cortex a significant increase was observed only in WT, with strong trend in C*3*^*−/−*^ (Sham vs. P-TBI; WT: *p* = 0.022; *C3*^*−/−*^; *p* = 0.176), with a decrease in *CCL2* expression between *CD14*^*−/−*^ and *C3*^*−/−*^*/CD14*^*−/−*^ compared with WT P-TBI (*CD14*^*−/−*^: *p* = 0.063; *C3*^*−/−*^*/CD14*^*−/−*^: *p* = 0.038, despite the non-significant omnibus results: *p* = 0.467). **C, D** RT-PCR analysis of *CCL2* expression in ipsi- and contralateral hippocampus post sham and P-TBI revealed a significant increase in all genotypes in the ipsilateral hippocampus (Sham vs. P-TBI; WT: *p* = 0.005; *C3*^*−/−*^: *p* = 0.009; *CD14*^*−/−*^: *p* = 0.042; *C3*^*−/−*^*/CD14*^*−/−*^: *p* < 0.001), with no differences in the P-TBI between the genotypes. In the contralateral hippocampus a significant increase was observed only in WT (Sham vs. P-TBI; WT: *p* = 0.001), with a decrease in *CCL2* expression in all genotypes compared with WT P-TBI (*C3*^*−/−*^: *p* = 0.002; *CD14*^*−/−*^: *p* = 0.003; *C3*^*−/−*^*/CD14*^*−/−*^: *p* = 0.0005, despite the non-significant omnibus results: *p* = 0.098). **E, F** RT-PCR analysis of *CCL2* expression in ipsi- and contralateral striatum post sham and P-TBI revealed an increase in WT, and *C3*^*−/−*^*/CD14*^*−/−*^ in the ipsilateral striatum (Sham vs. P-TBI; WT: *p* = 0.057; *C3*^*−/−*^*/CD14*^*−/−*^: *p* = 0.057), with a trend to decrease expression in *C3*^*−/−*^ compared to WT P-TBI (*p* = 0.064). In the contralateral striatum a strong trend was observed only in the *CD14*^*−/−*^ (Sham vs. P-TBI; *CD14*^*−/*−^: *p* = 0.166), with no differences between the genotypes. Data shown as bar plots with individual data points. Group size: *N* = 6–11. Exact* p*-values are reported, significances are shown in bold

We sought an independent confirmation of the region-specific roles of C3 and CD14 in the neuroinflammatory response to P-TBI by assessing the expression of CCL2 across genotypes and brain structures. Mixed effects analysis revealed a significant effect (or strong trend) in sham vs. P-TBI in all regions (ipsilateral cortex: *p* < 0.001; contralateral cortex: *p* = 0.069; ipsilateral hippocampus: *p* < 0.001; contralateral hippocampus: *p* = 0.081; ipsilateral striatum: *p* = 0.049; contralateral striatum: *p* = 0.012). The prespecified pairwise comparison showed that for the ipsilateral cortex, CCL2 was upregulated in all genotypes (WT: *p* = 0.059; *C3*^*−/−*^: *p* = 0.041; *CD14*^*−/−*^: *p* = 0.059; *C3*^*−/−*^*CD14*^*−/−*^: *p* = 0.01; Fig. 8A), for the contralateral cortex however, an upregulation of CCL2 was observed only in WT (WT: *p* = 0.022; Fig. 8B). For the ipsilateral hippocampus, an upregulation of CCL2 was found all genotypes (WT: *p* = 0.005; *C3*^*−/−*^: *p* = 0.009; *CD14*^*−/−*^: *p* = 0.042; *C3*^*−/−*^*CD14*^*−/−*^: *p* < 0.001; Fig. 8C), for the contralateral hippocampus, an upregulation of CCL2 was only found in WT animals (WT: *p* = 0.001; Fig. 8D). In the ipsilateral striatum, the pairwise comparison showed a strong trend to upregulation of CCL2 in the WT and combined *C3*^*−/−*^*CD14*^*−/−*^ animals (WT: *p* = 0.057; *C3*^*−/−*^*CD14*^*−/−*^: *p* = 0.057; Fig. 8E), for the contralateral striatum, no CCL2 upregulation was observed (Fig. 8F). Suggesting that CCL2 displays a region-specific dependency on C3 and CD14, similar to that of TNF.

For the contrast P-TBI across genotypes, we observed no significant decreases in the ipsilateral side, with a trend in the striatum for *C3*^*−/−*^ (*p* = 0.064). For the contralateral side, a significant decrease was observed in the cortex for *CD14*^*−/−*^: and *C3*^*−/−*^*CD14*^*−/−*^ (*p* = 0.042 and *p* = 0.038). For the hippocampus, a significant decrease was observed in all genotypes. For the striatum no differences were observed.

Thus, the *CCL2* response to P-TBI displays similarities to *TNF* with a stronger CD14 dependency in cortex, a C3 dependency in striatum and a C3 and CD14 dependency in hippocampus. For the ipsilateral site, a dependency of C3 was observed in the striatum only where the loss of CD14 resulted in a pro-inflammatory response that overcame the C3 loss.

### C3 and CD14 do not affect systemic haemodynamic or organ damage in P-TBI

Having observed the differential effect of C3 and CD14 on the induction of a cerebral neuroinflammatory response in P-TBI, we reasoned that the central effects may actually be explained by differences in systemic inflammatory responses and peripheral organ damage. Thus, we considered the haemodynamic profiles in WT, *C3*^*−/−*^, *CD14*^*−/−*^ and in *C3*^*−/−*^*CD14*^*−/−*^ animals during the HS/resuscitation phase of the P-TBI (Suppl. Figure 3 A). Plasma levels of C3b confirmed the genotype of mice, with no differences upon P-TBI (Suppl. Figure 3B). Arterial pressure trends were comparable across the four genotypes in both sham groups as well as P-TBI groups (Suppl. Figure 3 C–E). As expected, heart rate showed a significant increase at around 180 min into the procedure between sham and P-TBI (WT sham vs. WT P-TBI: *p* < 0.05 at 155, 180–185 and 195–205 min; Suppl. Figure 3 F). When considering the different genotypes, a small significant increase was observed between WT and *CD14*^*−/−*^ mice around 180 min into the procedure in both sham and P-TBI groups (WT sham vs. *CD14*^*−/−*^ sham: *p* < 0.05 at 180, 195–205 min; WT P-TBI vs. *CD14*^*−/−*^ P-TBI: *p* < 0.05 at 180–185 and 195–205 min; Suppl. Figure 3G, H).

Furthermore, we explored a battery of plasma damage markers related to specific organs, including brain (astroglial, GFAP), lungs (CC16), liver (ALT), pancreas (Lipase), intestines (I-FABP) and vasculature (syndecan), or systemic inflammatory markers, like: PAI-1, IL-6 and C5a. All damage markers (except for C5a, Suppl. Figure 3Q) were significantly increased upon P-TBI in WT mice (Suppl. Figure 3I-P), but were largely unaffected by the loss of C3 and/or CD14: no effect was found in glial damage, liver damage, kidney damage and systemic inflammation (C5a, IL-6) and only isolated effects on the intestine (I-FABP in *C3*^*−/−*^; Suppl. Figure 3 M) and the vascular system (Syndecan in *C3*^*−/−*^; Suppl. Figure 3 N).

Thus, the absence of C3 and/or CD14 does not result in significant improvement in hemodynamic parameters, tissue damage or inflammation upon P-TBI, strongly suggesting that the observed differences at the cerebral level are due to focal alterations in the neuroinflammatory response rather than consequences of changes in systemic inflammation.

### The expression pattern of C3 and CD14 across brain structures upon P-TBI is associated with the differential requirement for the induction of inflammatory cytokines

**Fig. 9:**
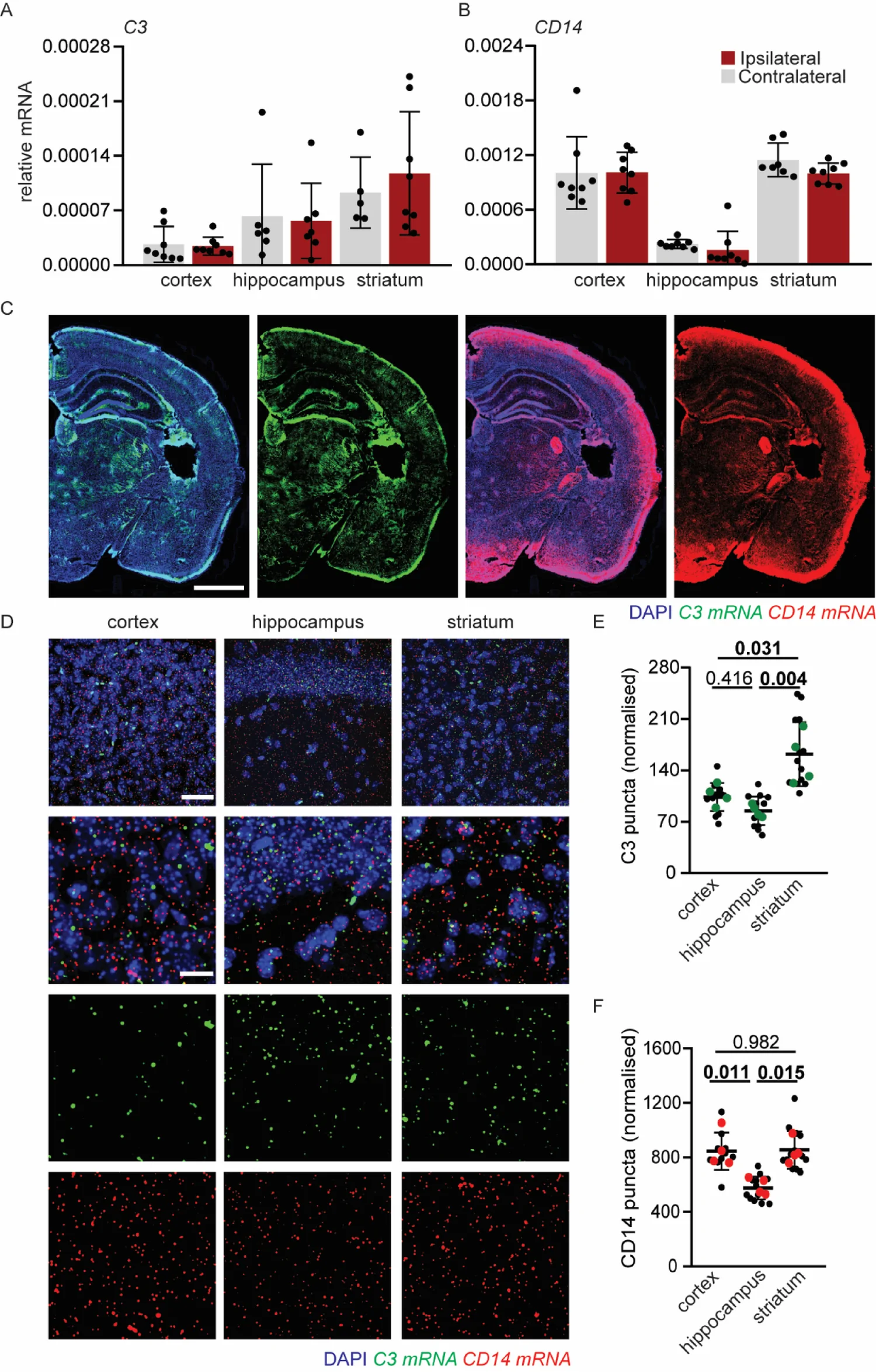
Differential pattern of C3 and CD14 expression across brain regions. **A, B** RT-PCR analysis of *C3* and *CD14* expression in WT cortex, hippocampus and striatum revealed low, intermediate and high expression of *C3* in cortex, hippocampus and striatum respectively. For *CD14*, a high, low, high expression in cortex, hippocampus and striatum was observed. **C–F** Single mRNA in situ hybridization of *C3* and *CD14* expression in cortex. *C3* showed highest expression in striatum, compared to cortex and hippocampus (cortex vs. striatum: *p* = 0.031; hippocampus vs. striatum: *p* = 0.004). *CD14* showed highest expression in cortex and striatum, and lowest expression in hippocampus (cortex vs. hippocampus: *p* = 0.011; hippocampus vs. striatum: *p* = 0.015). Data shown as bar plots with individual data points or dot plots as dot plots with single sections depicted as black dots and single mice as coloured dots (green/red). Scale bar overviews: 200 and 50 μm; insert: 10 μm. Group size: expression data *N* = 5–8; RNAscope data *N* = 4. Exact* p*-values are reported, significances are shown in bold

To further investigate the differential effect of C3 and CD14 in the induction of *TNF* and *CCL2* in the cortex vs. striatum vs. hippocampus, we first investigated the expression of *C3* and *CD14* mRNA by qPCR. Expression of *C3* was very limited but revealed a striking pattern, being weakest in the cortex and strongest in the striatum (Fig. 9A). Conversely, *CD14* expression was substantially higher than *C3* in absolute terms, and it was comparable in the striatum and in the cortex but was substantially weaker in the hippocampus (Fig. 9B).

We confirmed this expression pattern using single-molecule in situ hybridization. The density of *C3* mRNA was higher in the striatum than in the cortex or the hippocampus on average (cortex vs. striatum: *p* = 0.031; hippocampus vs. striatum: *p* = 0.004; Fig. 9D, E). However, *C3* mRNA distribution was not homogeneous: in the cortex, the expression was concentrated in the layer IV and V, where in the hippocampus it overlapped the neuronal outlines of dentate gyrus and CA fields (Fig. 9C). On the other hand, the density of *CD14* was comparable in the cortex and striatum (in agreement with qPCR data) and weaker in the hippocampus (cortex vs. hippocampus: *p* = 0.011; hippocampus vs. striatum: *p* = 0.015; Fig. 9D, F); *CD14* was diffusely expressed through the cortex and the striatum, whereas in the hippocampus was concentrated in the stratum oriens and stratum moleculare (Fig. 9C).

Thus, the expression data suggest a partial association between regional C3 and CD14 expression levels and their differential involvement in neuroinflammatory activation after P-TBI. The cortex appears more sensitive to CD14, despite its high expression in both the cortex and striatum. In contrast, the striatum, which also exhibits high C3 expression, depends exclusively on C3 to drive diffuse neuroinflammation following P-TBI, even when CD14 expression is robust.

### Loss of C3 and CD14 reduces overall neuronal injury and shapes structure-specific neuronal immediate-early genes induction in P-TBI

**Fig. 10:**
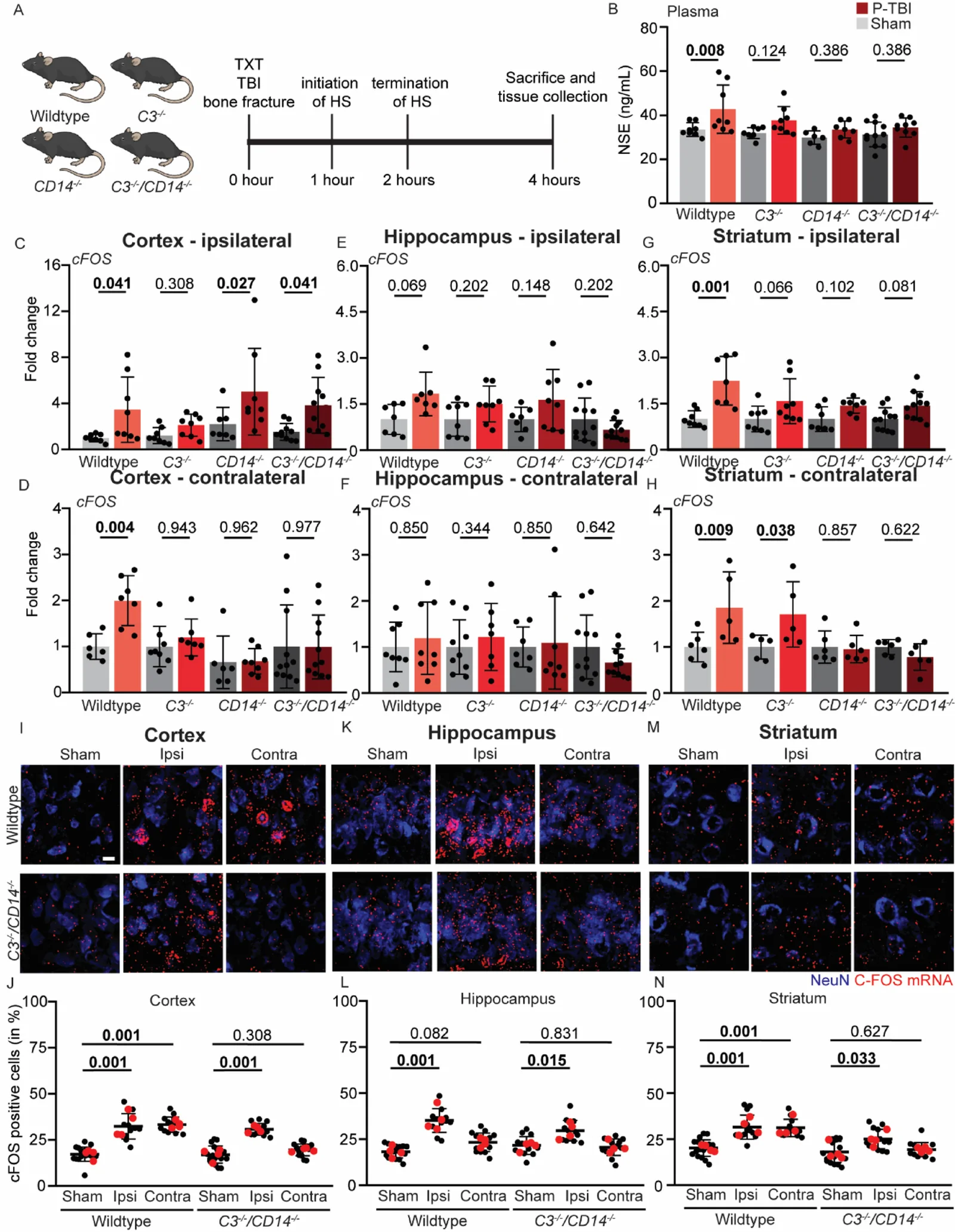
C3 and CD14 regulate plasma injury markers and *cFOS* expression patterns across brain regions. **A** Experimental design of polytrauma and TBI (P-TBI) in wildtype, *C3*^*−/−*^, *CD14*^*−/−*^ and *C3*^*−/−*^*/CD14*^*−/−*^ mice, investigating neuronal injry responses. **B** ELISA analysis of neuronal injury marker NSE reveals significant upregulation upon P-TBI in plasma of WT mice (*p* = 0.008) though not in *C3*^*−/−*^, *CD14*^*−/−*^ and *C3*^*−/−*^*/CD14*^*−/−*^ mice. Between genotype analysis revealed significant differences in *CD14*^*−/−*^ and *C3*^*−/−*^*/CD14*^*−/−*^ in comparison to WT mice (*CD14*^*−/−*^: *p* = 0.008; *C3*^*−/−*^*/CD14*^*−/−*^: *p* = 0.008). **C-D.** RT-PCR analysis of *cFOS* expression levels in in ipsi- and contralateral cortex post sham and P-TBI revealed an increase in WT, *CD14*^*−/−*^ and *C3*^*−/−*^*/CD14*^*−/−*^ in the ipsilateral cortex (Sham vs. P-TBI; WT: *p* = 0.041; *CD14*^*−/−*^: *p* = 0.027; *C3*^*−/−*^*/CD14*^*−/−*^: *p* = 0.041), with no differences between the genotypes and WT P-TBI. For the contralateral cortex a significant increase was only observed in WT (*p* = 0.004), with a decrease in cFOS expression levels in all genotypes compared to WT P-TBI (*C3*^*−/−*^: *p* = 0.014; *CD14*^*−/−*^: *p* < 0.001; *C3*^*−/−*^*/CD14*^*−/−*^: *p* = 0.002). **E, F** RT-PCR analysis of *cFOS* expression levels in in ipsi- and contralateral hippocampus post sham and P-TBI revealed an increase in WT in the ipsilateral hippocampus (though not significant; Sham vs. P-TBI; WT: *p* = 0.069), with a decrease between *C3*^*−/−*^*/CD14*^*−/−*^ and WT P-TBI (*p* = 0.001; despite the non-significant omnibus results: *p* = 0.056). For the contralateral hippocampus no differences were observed. **G, H** RT-PCR analysis of *cFOS* expression levels in in ipsi- and contralateral striatum post sham and P-TBI revealed an increase in WT, with strong trends in *C3*^*−/−*^, *CD14*^*−/−*^ and *C3*^*−/−*^*/CD14*^*−/−*^ in the ipsilateral cortex (Sham vs. P-TBI; WT: *p* < 0.001; *C3*^*−/−*^: *p* = 0.066; *CD14*^*−/−*^: *p* = 0.102; *C3*^*−/−*^*/CD14*^*−/−*^: *p* = 0.081), between genotype analysis revealed differences between the genotypes and WT P-TBI (*C3*^*−/−*^: *p* = 0.011; *CD14*^*−/−*^: *p* = 0.007; *C3*^*−/−*^*/CD14*^*−/−*^: *p* = 0.004). For the contralateral striatum a significant increase was observed in WT and *C3*^*−/−*^ (sham vs. P-TBI; WT: *p* = 0.009; *C3*^*−/−*^: *p* = 0.038), with a decrease in *cFOS* expression levels in *CD14*^*−/−*^ and *C3*^*−/−*^*/CD14*^*−/−*^ compared to WT P-TBI (*CD14*^*−/−*^: *p* = 0.002; *C3*^*−/−*^*/CD14*^*−/−*^: *p* = 0.001). **I–N** Single mRNA in situ hybridization of *cFOS* in NeuN+ neurons revealed significant increase of *cFOS*+ neurons in ipsi- and contralateral cortex (WT sham vs. WT P-TBI ipsi: *p* < 0.001; WT sham vs. WT P-TBI contra: *p* < 0.001), ipsilateral hippocampus (WT sham vs. WT P-TBI ipsi: *p* < 0.001; WT sham vs. WT P-TBI contra: *p* = 0.082) and bilateral striatum (WT sham vs. WT P-TBI ipsi: *p* = 0.001; WT sham vs. WT P-TBI contra: *p* = 0.001) of WT mice. An increase of *cFOS*+ neurons was observed only in ipsilateral cortex (*p* < 0.001), hippocampus (*p* = 0.015) or striatum (*p* = 0.033) for *C3*^*−/−*^*/CD14*^*−/−*^ mice, with no differences in contralateral side. Data shown as dot plots with single sections depicted as black dots and single mice as coloured dots (green). Scale bar: 10 μm. Group size: *N* = 3–4. Exact* p*-values are reported, significances are shown in bold

Having described the structure-specific neuroinflammatory response to P-TBI and the differential sensitivity to the loss of C3 and/or CD14, we set out to investigate how the modified early neuroinflammatory response would impact neuronal viability and large-scale neuronal activity. First, we explored the overall neuronal injury using the plasma levels of Neuron-Specific Enolase (NSE; [27, 28]). Mixed effects analysis revealed a significant effect in sham vs. P-TBI (*p* = 0.002; Fig. 10B). Prespecified pairwise comparison across genotypes revealed a significant increase of NSE in sham vs. P-TBI only in WT (WT: *p* = 0.008; Fig. 10B), whereas loss of C3 and/or of CD14 prevented NSE elevation (mixed effects for genotypes: *p* = 0.023); pairwise comparison revealed a significantly lower NSE in P-TBI *CD14*^*−/−*^ (*p* = 0.008) and *C3*^*−/−*^*CD14*^*−/−*^ (*p* = 0.008) animals compared to WT but only a non-significant trend in *C3*^*−/−*^ (*p* = 0.078; Fig. 10B).

Second, we investigated the large-scale neuronal activity by monitoring the expression of immediate early genes [29, 30] *cFOS*, *FosB*, and *ARC*. We found that both *cFOS* and *FosB* were increased in both ipsilateral (*cFOS*: *p* = 0.029; *FosB*: *p* = 0.023) and contralateral (*cFOS*: *p* = 0.002; *FosB*: *p* = 0.005) cortex, whereas *ARC* was not upregulated (Suppl. Figure 4 A-B). We further quantified *FOS* expression levels across brain regions and genotypes. Mixed effects analysis revealed a significant effect of P-TBI in all regions (ipsilateral cortex: *p* = 0.001; ipsilateral hippocampus: *p* = 0.024; ipsilateral striatum: *p* < 0.001; contralateral striatum: *p* = 0.015), except contralateral cortex and hippocampus (cortex: *p* = 0.091; hippocampus: *p* = 0.459). Pairwise comparison showed that for the ipsilateral cortex, *cFOS* was equally upregulated in WT, *CD14*^*−/−*^ and *C3*^*−/−*^*CD14*^*−/−*^ (WT: *p* = 0.041; *CD14*^*−/−*^: *p* = 0.027; *C3*^*−/−*^*CD14*^*−/−*^: *p* = 0.041; Fig. 10C) but, most notably, not in the *C3*^*−/−*^. In the contralateral cortex the upregulation of *cFOS* was observed only in WT (WT: *p* = 0.004; Fig. 10D) and abolished by the loss of C3 and/or CD14. In the ipsilateral hippocampus, the pairwise comparison revealed no significant increase in P-TBI vs. Sham in any genotype (trend only for WT: *p* = 0.069; Fig. 10E); no significant increase was observed in contralateral hippocampus (Fig. 10F). Interestingly, whereas in the ipsilateral striatum, the pairwise comparison showed a significant upregulation of *cFOS* in the WT (*p* < 0.001; Fig. 10G), for the contralateral striatum, the pairwise comparison showed a significant increase in WT and *C3*^*−/−*^ (WT: *p* = 0.009; *C3*^*−/−*^: *p* = 0.038; Fig. 10H).

In agreement with the P-TBI vs. Sham, the contrast of P-TBI groups revealed no differences across genotypes in ipsilateral cortex but significant differences in contralateral cortex, as well as, in the case of striatum, significant differences between P-TBI in WT vs. P-TBI in animals lacking C3 and/or CD14 in ipsilateral and only for animals lacking CD14 in the contralateral side (Fig. 10C–H; full statistical description in Supplementary file descriptive statistics).

In addition to *cFOS*, we quantified *FosB* across brain regions and genotypes. Mixed effect analysis revealed a significance in cortex, ipsilateral hippocampus and a trend in ipsilateral striatum (ipsilateral cortex: *p* = 0.001; contralateral cortex: *p* = 0.004; ipsilateral hippocampus: *p* = 0.004; contralateral hippocampus: *p* = 0.056; ipsilateral striatum: *p* = 0.059; contralateral striatum: *p* = 0.259). The pairwise comparison revealed a significant increase in the ipsilateral cortex for *CD14*^*−/−*^ and *C3*^*−/−*^*CD14*^*−/−*^, with a trend in WT and *C3*^*−/−*^ (WT: *p* = 0.082; *C3*^*−/−*^: *p* = 0.112; *CD14*^*−/−*^: *p* = 0.002; *C3*^*−/−*^*CD14*^*−/−*^: *p* = 0.042; Suppl. Figure 4 C). For the contralateral cortex, a significant increase was only observed in WT (*p* < 0.001; Suppl. Figure 4D). For the hippocampus, a significant upregulation was observed only in ipsi- and contralateral WT (ipsi: *p* = 0.007; contra: *p* = 0.015 Suppl. Figure 4E, F). In case of the striatum, a significant increase was only found in ipsi- and contralateral WT (ipsi: *p* = 0.021; contra: *p* = 0.024; Suppl. Figure 4G, H).

The contrast of P-TBI showed no differences across genotypes in ipsilateral cortex, but significant differences in contralateral cortex. Ipsilateral hippocampus revealed significant differences in mice lacking both C3 and CD14, and only for mice lacking CD14 in contralateral hippocampus. For the striatum, the ipsilateral side showed significant differences in *CD14*^*−/−*^ and all genotypes for the contralateral striatum (Suppl. Figure 4 C–H).

Third, we employed the in-situ hybridization of for *cFOS* mRNA and the co-immunostaining for NeuN to ensure that the Fos signal observed in expression studies corresponded to actual neuronal responses. We quantified single *cFOS* mRNA molecules in neurons from histological sections of cortex, hippocampus and striatum from *C3*^*−/−*^*CD14*^*−/−*^ animals. Mixed effect analysis showed a significant upregulation in the fraction of *cFOS*+/NeuN+ cells in the cortex, hippocampus and striatum (Cortex: *p* < 0.001; hippocampus: *p* < 0.001; striatum: *p* < 0.001). In detail, the prespecified pairwise comparison of P-TBI vs. Sham for WT animals revealed a significant increase in both ipsi- and contralateral cortex (ipsi: *p* < 0.001; contra: *p* < 0.001; Fig. 10I, J), ipsilateral hippocampus (ipsi: *p* < 0.001; Fig. 10K, L) and ipsi- and contralateral striatum (ipsi: *p* = 0.001; contra: *p* = 0.001; Fig. 10M, N). On the other hand, no such increase in NeuN+/*cFOS* mRNA + was observed in *C3*^*−/−*^*CD14*^*−/−*^ in contralateral cortex (ipsi: *p* < 0.001; contra: *p* = 0.308; Fig. 10I, J), contralateral hippocampus (ipsi: *p* = 0.015; contra: *p* = 0.831; Fig. 10K, L) nor contralateral striatum (ipsi: *p* = 0.033; contra: *p* = 0.627; Fig. 10M, N), once again demonstrating that the increase in neuronal activity occurring after P-TBI is prevented by the loss of C3 and CD14.

In contrast to the inflammatory effects, this data reveals that neuronal activity shows a more C3 dependent effect in the cortex, with a CD14 dependency in striatum. For the hippocampus, both C3 and CD14 are mediating *cFOS* levels.

## Discussion

**Fig. 11:**
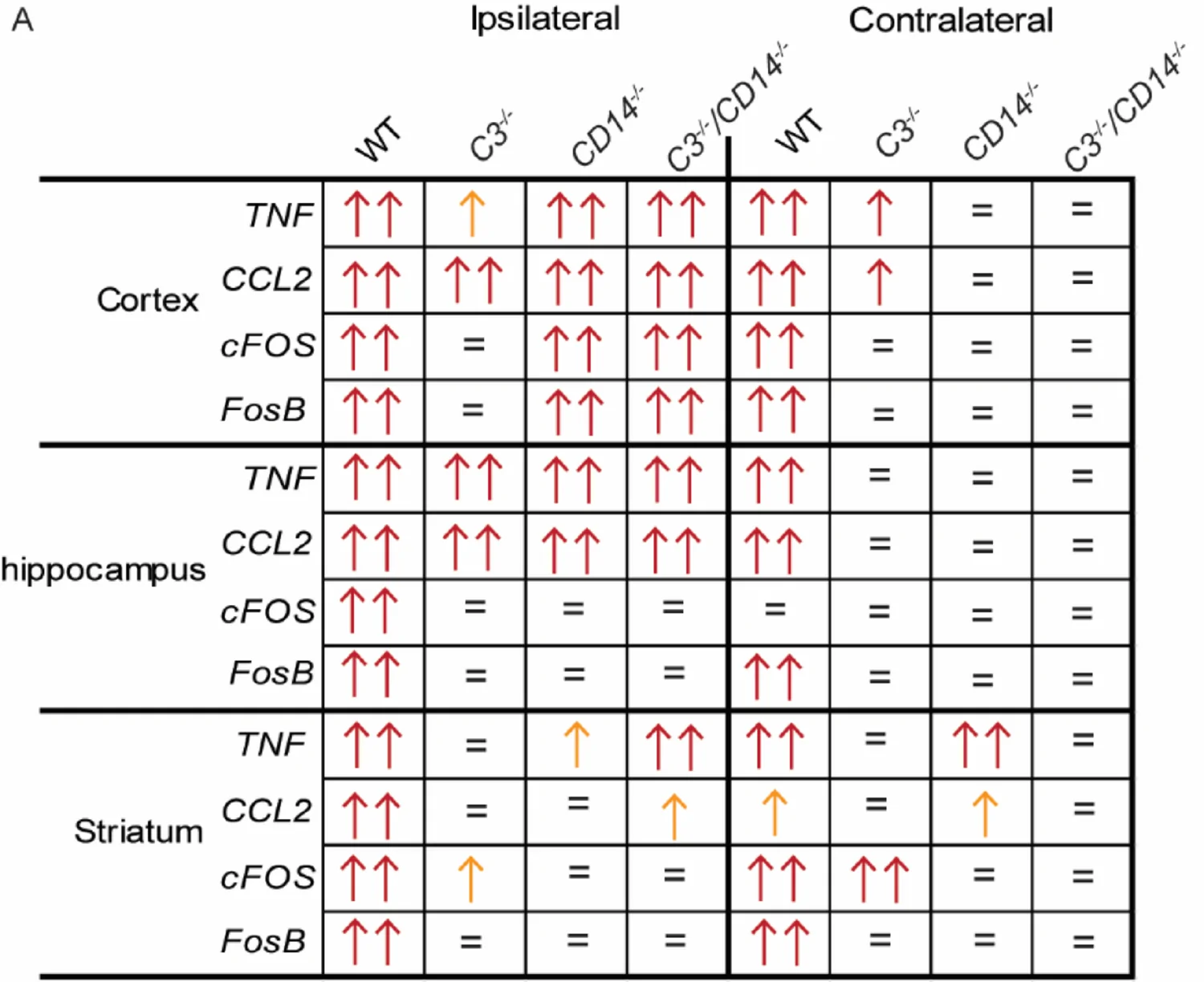
Summary of the region-specific and genotype specific findings. **A** Significant increase (*p* < 0.05; two red arrow), trend to increase (0.05 < *p* < 0.15; one orange arrow) and no difference (*p* > 0.15; equal) of transcriptional responses, *TNF*, *CCL2*, *cFOS* and *FosB* across all brain regions (cortex, hippocampus and striatum) in all genotypes (WT, *C3*^*−/−*^, *CD14*^*−/−*^ and *C3*^*−/−*^*/CD14*^*−/−*^)

**Fig. 12:**
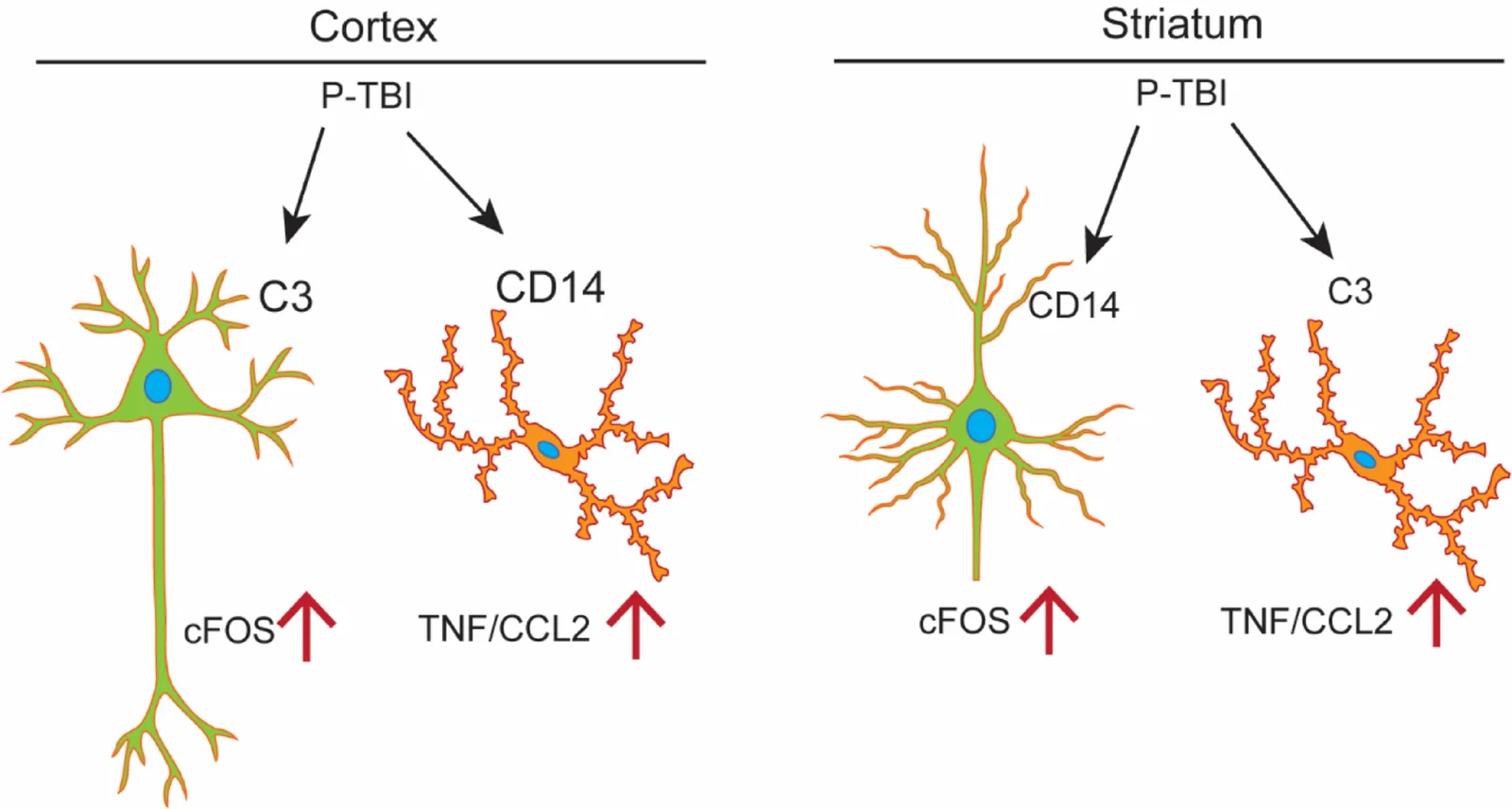
Graphical abstract. Cortex shows a neuronal C3 dependency for the expression of *cFOS*, and a microglial CD14 dependency for the expression of *TNF*/*CCL2* post P-TBI. Striatum shows a neuronal CD14 dependency for the expression of *cFOS* and a microglial C3 dependency for the expression of *TNF*/*CCL2* post P-TBI

Our investigation of the P-TBI model, including bone fracture, thorax trauma, HS and mild TBI, has revealed an early and diffuse induction of inflammatory cytokines across the brain, highlighted by the upregulation of *TNF*, *CCL2* and *IL-1β* and the appearance of reactive microglia. Contrary to our hypothesis, systemic inflammation and organ damage is not suppressed in mice lacking C3 or CD14, although in animals the cerebral inflammatory response is prevented or reduced and the microglial reactivity is abolished. The impact of C3 and/or CD14 appears to be different across brain structures, in particular in the hemisphere contralateral to the injury: whereas *TNF* and *CCL2* induction in cortex is dependent on CD14, it is dependent on both C3 and CD14 in hippocampus and it is dependent on C3 alone in the striatum. In the ipsilateral hemisphere, the induction of *TNF* and *CCL2* are not modified by the loss of C3 and/or CD14, indicating substantial redundancy in the early innate-immunity response. Conversely, the neuronal excitation response, as assessed by the expression of immediate-early genes *cFOS* and *FosB*, follows an almost opposite pattern, being sensitive to C3 (or to both C3 and CD14) in the cortex but sensitive to CD14 in the striatum (ipsi- and contralateral (these patterns are depicted in Fig. 11). The pattern of expression of C3 and CD14 are suggestive of a possible link between expression level and requirement for the early response microglia, but not of neurons, in P-TBI (Fig. 12).

Our finding of a rather diffuse induction of inflammatory cytokines across the brain, on which the focal induction by TBI is superimposed, are cross-validated by previous findings showing a substantial degree of neuroinflammation occurring upon peripheral injury [31], polytrauma [32, 33], or more broadly, during systemic inflammatory conditions [34] such as in sepsis [15]. In our model, as well as in previous reports [32], the cerebellum remains somehow unaffected. Interestingly, we identified a significant upregulation of *CCL2* in the cortex and hippocampus, but an actual downregulation was previously reported [32]; however, we considered an earlier timepoint (4 h vs. 6 h) and our polytrauma was less severe (not including peripheral limb ischemia or amputation) than the previous one, suggesting that the neuroinflammatory response to P-TBI is context-dependent. Furthermore, *CCL2* and *TNF*, has been reported to be upregulated in both ipsi- and contralateral cortex and hippocampus following TBI, in a controlled cortical impact (CCI) and fluid percussion model [35, 36], however those models are more severe than the closed-head weight-drop model used here. The neuroinflammatory response after P-TBI is also structure-dependent: the response occurring in the striatum lacks the *CXCL2* upregulation observed in the cortex and hippocampus; this finding is in agreement with the distinct segregation of striatal samples in previously reported P-TBI datasets [32] and underscore a model in which the neuroimmune milieu is distinct and unique in different structures.

In the context of the P-TBI, the inflammatory cascades appear to display a different degree of redundancy in the focal injury and in the widespread area of involvement. This difference is clearly seen in the sensitivity to the lack of C3 and/or CD14: the cytokine induction in the site of mild TBI is resistant to the loss of C3 and/or CD14, indicating a substantial redundancy in the DAMP signaling, whereas the cytokine induction in the apparently non-injured brain remains sensitive, to C3 in striatum but to CD14 loss in cortex, whereas both molecules are necessary in hippocampus. A similar redundancy of DAMP signaling may be preventing the loss of C3 and CD14 from affecting peripheral tissue damage and systemic inflammation.

Complement activation takes place within minutes after polytrauma [8] and the release of multiple biologically active proteolytic fragments from C3 and C5 drives a massive activation of innate immunity cells, such as neutrophils and macrophages [9], and the release of other inflammatory cytokines [37]. Complement activation is critical for secondary damage to kidney and lungs in polytrauma [38, 39]. CD14, on the other hand, acts as a co-factor contributing to the signaling of multiple TLR, which recognize not only pathogens-associated molecules (PAMPs) but also DAMPs [4]. CD14 has been implicated in the response to polytrauma and to secondary injury to kidney, intestine and liver [5, 40]. Our findings of abolished microglial reaction to polytrauma in mice lacking C3 and CD14 are aligned with previously reported beneficial effects in isolated TBI obtained in *C3*^*−/−*^ animals or upon complement depletion [18, 41] and are in line with the reduced microglial inflammation observed in *CD14*^*−/−*^ animals [42]. However, the study of single- and double-KO animals reveals a larger dependency on CD14 in the cortex, C3 in the striatum and the dependency on either C3 or CD14 in the hippocampus. which could be only in part correlated with the expression pattern of these molecules. Interestingly, simultaneous loss of C3 and CD14 does not ameliorate the damage to any other organ considered (in particular lungs, liver, pancreas, and vasculature) in our polytrauma model with HS, although beneficial effects have been reported upon combined C5 and CD14 inhibition in a porcine polytrauma model [5]. Thus, both C3 and CD14 are dispensable for the systemic inflammatory response to polytrauma in the present murine model, likely because of the redundant activation of inflammatory pathways by the massive release of DAMP but appear necessary to mediate the cerebral inflammatory response in these conditions.

The contrast of brain samples from WT animals and from single *C3*^*−/−*^ or *CD14*^*−/−*^ or from double *C3*^*−/−*^*CD14*^*−/−*^ KO reveals that while *CCL2* and *TNF* induction in the cortex depends largely on CD14, both cytokines are mainly dependent on C3 in striatum. Actually, in striatum we also detected some anti-inflammatory role of CD14, since *TNF* and *CCL2* mRNA upregulation is abolished in *C3*^*−/−*^ but is re-instated in *C3*^*−/−*^*CD14*^*−/−*^ (Figs. 7I and 8E), indicating an opposite effect of the loss of C3 and CD14; these findings are in agreement with previously reported pro- and anti-inflammatory effects of CD14 loss in the microglia [42]. Our findings extend the previously reported role of C3 in driving striatal neuroinflammation upon *Staphylococcus* infection [43] as well as of its role in mediating microglial reactivity in a hypoperfusion model [44]. In contrast, the response to LPS, which requires CD14, has been previously shown to be different in the cortex and striatum [45], with substantially higher neurotoxicity observed in the striatum. More broadly, sensitivity to LPS has been shown to exhibit strong regional variations across the brain, with *TNF* induction resulting up to five times higher in the cortex than in subcortical structures [46]. Overall, the differential effects of C3 and/or CD14 loss cannot be fully explained by their expression patterns alone, highlighting region-specific regulatory mechanisms.

We further explored the effect of P-TBI on neuronal excitation (as measured by the proxy immediate-early genes c*FOS* and *FosB*) and the mechanistic involvement of C3 and CD14. We noticed the upregulation of c*FOS* and *FosB* in almost any location explored (in agreement with previous evidence: [29, 47]), coherent with the effect of P-TBI on large-scale neuronal networks (also shown in other systemic inflammatory conditions and isolated TBI models; [48]). Interestingly, *FOS* induction was affected by the loss of C3 and/or CD14 in a different way then *TNF* and *CCL2* induction: CD14 is required for *cFOS* induction in neurons in the striatum, whereas C3 is necessary for cytokine induction. This difference is in line with previous reports indicating a major role for CD14 in mediating the neurotoxicity of LPS in the striatum [42, 45]. It must be noted that whereas *TNF* and *CCL2* mRNA are largely (although not uniquely) induced in microglial cells, the count of *cFOS* mRNA is related to neuronal responses. Thus, simultaneous interference with C3 and CD14 may not only target inflammatory responses throughout different structures but also target different cellular subpopulations. This redundancy is compatible with the effect observed on the neuronal marker NSE, which may report both neuroinflammatory and excitotoxic damage to neurons, and whose elevation was decreased by either C3 or CD14 loss.

The present work displays some unavoidable limitations. First, we considered a single, early timepoint for the tissue sampling; because of the high severity of the injury, criteria set in place to avoid unnecessary suffering of the animals did not allow for longer time courses. It is anticipated that the neuroimmune trajectory of the P-TBI may then evolve toward a phase of resolution, either complete, although in more severe P-TBI models, this is not observed (Rowe et al., 2024) or most likely to a permanent post P-TBI inflammatory state. Second, we focused our analysis on microglial cells, since they are highly sensitive to inflammatory stimuli and therefore constitute suitable readouts for interventions. Astrocytes, resident macrophages, pericytes and even neurons can contribute to the cytokine milieu (Janova et al., 2016). The discrepancy between microglia-selective cytokine expression and whole-tissue expression. e.g., the effect of the loss of both C3 and CD14 is detected at microglial level in ipsilateral cortex but not at whole-tissue level, points toward additional contributions to cytokine induction that are not C3 and/or CD14 sensitive and may not respond to therapeutic interventions on complement.

Taken together, our data support the acute onset of neuroinflammation in the context of P-TBI, similar to other systemic inflammatory response conditions (Chung et al., 2023). The profile of the neuroimmune activation is uniquely dependent on cerebral C3 and, to a lesser extent, on cerebral CD14, whereas for systemic inflammation in P-TBI in this model their role appears dispensable. Interventions on C3 and/or CD14 may therefore be titrated depending on the occurrence of a cerebral focal injury or diffuse encephalopathy.

## Methods

### Experimental animals and genotypes

Mouse polytrauma experiments have been approved by the animal oversight committee at Ulm University and by the corresponding authorities (Regierungspräsidium in Tübingen; Reg. 1480). Male mice aged 14–16 weeks of four different strains (wild-type: C57BL6; *C3*^*−/−*^: B6.129S4-C3tm1Crr/J; *CD14*^*−/−*^: B6.129S4-Cd14tm1Frm/J [49] and *C3*^*−/−*^*/CD14*^*−/−*^: B6.129S4-C3tm1Crr/ Cd14tm1Frm/J) were generated by Prof. Egil Lien (UMASS, Boston, MA) and provided by Prof. Tom Eirik Mollnes (University of Oslo, Norway), additional wild-type mice (C57BL6) were purchased for Janvier-Labs (France). C57BL6 wildtype mice are considered the recommended controls, by the Jackson Laboratory, for both *C3*^*−/−*^ (https://www.jax.org/strain/003641) and *CD14*^*−/*^ mice (https://www.jax.org/strain/003726).

### Polytrauma model

The polytrauma and haemorrhagic shock (P-TBI) murine trauma model, consisting of blunt thorax trauma (TXT), traumatic brain injury (TBI), closed unilateral diaphyseal femoral fracture and a haemorrhagic shock (HS) was performed as previously reported [50, 51]. Briefly, mice of each genotype were randomized either into the P-TBI group or sham surgery group, followed by buprenorphine administration (0.05 mg/kg, subcutaneously) and sevoflurane anaesthesia (3.0% in 97% O2). The bilateral blunt thorax trauma was performed using a well-defined blast wave. The traumatic brain injury was induced by closed head weight drop with a weight of 333 g falling from a height of 2 cm on the parietal bone. The closed diaphyseal femoral fracture was performed on the right leg by a weight dropping device of 50 g falling from a height of 120 cm. Directly after the polytrauma, the left femoral artery and the right jugular vein were catheterized. The HS was started 1 h after polytrauma induction, by withdrawing blood from the femoral artery until the mean arterial pressure (MAP) dropped to 30 ± 5 mmHg, which was maintained for an additional 1 h. Following the haemorrhagic shock the mice received a 4-fold volume resuscitation of the withdrawn blood volume, with isotonic electrolyte solution (Jonosteril, Fresenius Kabi, Germany) over 30 min. After the fluid therapy, mice that could not reach a MAP above 50 mmHg, received norepinephrine (0.5-2.0 ug/kg/min). Control mice (sham group) underwent the same treatments (analgesia, anaesthesia, femoral artery and jugular vein catheters, and application of 400 ul of isotonic electrolyte solution) without the induction of the TXT, TBI, femoral fracture and HS. During the whole procedure animals were kept under general anaesthesia (sevoflurane), analgesia (Buprenorphine) and monitoring of heart rate (HR) and MAP using a blood pressure analyser (DSI, St. Paul, Minn). The wildtype sham and P-TBI cohort used in Figs. 1 and 2 overlap with the wildtype cohort in Figs. 7 and 8. The individual analysis of each figure were performed in separate experimental setups, with all relevant experimental groups included and processed together within each setup.

### Blood and tissue sampling

Four hours after the induction of the P-TBI or sham surgery, mice were sacrificed followed by organ and blood harvesting for further processing. EDTA blood was drawn by cardiac puncture, followed by centrifugation for 5 min at 500 x g at 4 °C and storage at − 80 °C. The brain was dissected and snap frozen on dry ice, brain tissue was either used for protein isolation for antibody arrays or RNA isolation for RT-qPCR.

A cohort of mice (only wild-type and *C3*^*−/−*^*/CD14*^*−/−*^) were anesthetized with a lethal dose of ketamine (100 mg/kg) and Xylazine (16 mg/kg) in saline followed by transcardial perfusion with 25 mL of PBS and 50 mL of 4% PFA. Brain tissue was dissected and postfixed overnight at 4 °C in 4% PFA followed by washing in PBS and cryoprotection in 30% sucrose for 2 days. Brain samples were embedded in OCT (Tissue Tek) and stored at − 80 °C.

### Protein extraction and Antibody array analysis and bioinformatics

Proteins were isolated from the brain by adding RIPA buffer (150 mM NaCl, 50 mM Tris pH 7.6, 0.1% SDS, 1% Triton X-100, 1 mM EDTA), in a volume 4x the weight of the brain (weight: volume), protease and phosphatase inhibitors (Roche tablets) were added in excess, followed by homogenization of the tissue using a tissue grinder. The homogenate was sonicated for 2 × 2 s at 65%, followed by incubation for 30 min at 4 °C. The homogenate was centrifuged for 20 min at 10,000 x g, the supernatant was transferred to another tube and the pellet was discarded. The protein concentration was determined using the BCA assay (Thermo Fischer) according to the manufacturer’s instructions. Briefly, the homogenate was diluted 20x in RIPA buffer and added to the wells together with the standard in duplicates, 200ul of working reagent (50:1, reagent A: B) was added to each well and incubated for 30 min at 37 °C. The absorbance was measured at 562 nm with a plate reader. Sample concentration was determined by calculation from the standard curve.

Targeted cytokine proteomics was performed using the mouse inflammation array G1 from raybiotech (Raybiotech), according to manufacturers’ instructions. Briefly, glass slides were removed from − 20 °C and equilibrated for 30 min at RT. Slides were removed from the package and air-dried for another 2 h, followed by blocking by adding 100 ul sample diluent to each well for 30 min at RT. Buffer was decanted and 100 ul of sample (400 ug) was added to the wells and incubated overnight at 4 °C. Samples were decanted from the well and washed with 150 ul of 1x wash buffer I (diluted in dH2O) for 5 × 5 min at RT with gentle shaking, followed by another wash step with 150 ul of 1x wash buffer II (diluted in dH2O) for 2 × 5 min at RT with gentle shaking. Then, 80 ul of detection antibody cocktail was added to each well and incubated for 2 h at RT, followed by 5 × 5 min washing with wash buffer I and 2 × 5 min washing with wash buffer II. Then, 80 ul of Cy5 streptavidin (1:1000 in sample diluent) was added and incubated for 1 h at RT, followed by a 5 × 5 min washing with wash buffer I. The holders were disassembled and the glass slides were washed once for 15 min in an excess of wash buffer I (30 mL) and 5 min in an excess of wash buffer II (30 mL). After drying using a centrifuge at 1000 rpm for 5 min, the slides were scanned using a GenePix 4000B array scanner (Molecular Devices, LLC).

Antibody array analysis was performed using the R-studio version 4.3.1, using the limma package, utilizing linear models to model for experimental designs. Normalization within and between arrays was done using the cyclicloess method, to remove intensity-dependent biases between different samples and thereby making them more comparable. Then the arrays were corrected for any batch effects using the ComBat function, which estimates the mean and variance for each batch and adjusts the data accordingly. Then we proceeded towards differential expression, employing empirical Bayes methods that samples information across all proteins on the array, moderating the standard errors and improving the power to detect differential expression. The output was obtained using the topTable function to extract lists of differentially expressed proteins and their associated statistics like log fold-change,* p*-values, etc. as in Supplementary file 1.

### ELISA assay

The Enzyme-Linked Immunosorbent Assays (ELISA) were performed according to manufacturer’s instructions, with following dilutions, for Syndecan 1:10, I-FABP 1:100, GFAP 1:2 (LSbio), PAI-1 1:50, C5a 1:100 (R&D systems), LIPASE 1:5, C3b 1:1000 (Hycult), IL-6 1:2 (BD Bioscience), ALT 1:100 (Abcam), CC16 1:500 (Abbexa) and NSE 1:20 (Elabscience).

### RNA extraction and qPCR

Brain tissue was disrupted and homogenized in 1 ml of QIAzol (Qiagen, Germany). Following this, 200 µl of chloroform was added, and the mixture was vigorously vortexed for 15 s. After a 10-minute incubation at room temperature for phase separation, the homogenate was centrifuged at 12,000 × g for 10 min at 4 °C. The aqueous phase containing RNA was then transferred to a new tube. To precipitate the RNA, an equal volume of isopropanol was added to the aqueous phase, followed by a 10-minute incubation at room temperature. The mixture was centrifuged again at 12,000 × g for 10 min at 4 °C. The resulting RNA pellet was washed with 1 ml of 75% ethanol in DEPC-treated water and centrifuged at 8000 × g for 10 min at 4 °C. The ethanol was carefully removed, and the RNA pellet was allowed to air-dry completely before being reconstituted in 30 µl of RNase-free water. RNA concentration and purity were quantified using a NanoDrop spectrophotometer, and the RNA was stored at − 80 °C.

For reverse transcription, 1 µg of RNA was diluted to 40 µl with RNase-free water and 5 µl of random hexamers (Biomers, Germany) were added. After a 10-minute incubation at 70 °C for primer annealing, the mixture was cooled on ice. A master mix containing reverse transcriptase, RNase Inhibitor, dNTPs mix, and M-MLV RT 5x buffer was added to the RNA-primer solution. The reaction mixture was incubated at room temperature for 10 min, then at 42 °C for 45 min for cDNA synthesis. The reaction was terminated by heating at 99 °C for 3 min, then immediately placed on ice. The resulting cDNA was stored at − 20 °C.

Primers were designed using the Primer-BLAST tool from NCBI. Sequences from the NCBI GenBank database were input, and parameters were set for optimal PCR product size and primer melting temperature. Primers were synthesized by Biomers (Germany) and validated for specificity and functionality using test runs and controls. The detailed list of the primer sequences for each gene tested is reported in Supplementary Table 1. Quantitative RT-PCR (qRT-PCR) was conducted using the LightCycler 480 II system (Roche) with Power PCR TB Green PCR Master Mix (Takara, Japan). Each reaction included 2 µl of cDNA, 3 µl of primer mix, and 5 µl of TB Green in a 96-well plate. Samples were run in duplicate with GAPDH as an internal control. The threshold cycle value (Ct) was calculated and normalized using the Eq. 2 − ΔCt (ΔCt = Cttarget gene − CtGAPDH) to determine relative mRNA expression levels. Fold change was determined by comparing the experimental groups to the WT Sham group.

### Tissue sectioning and immunofluorescence staining

Brain tissue embedded in OCT were cryosectioned at 40 μm using a cryostat, followed by blocking of the tissue in blocking buffer (3% BSA, 0.3% triton-X100, 1× PBS) for 2 h at room temperature (RT). Sections were incubated for 48 h at 4 °C with primary antibodies (suppl. Table 1) diluted in blocking buffer, followed by 3 × 30 min washing in PBS at RT. Sections were incubated for 2 h at RT with secondary antibodies (Supplementary Table 1) diluted in blocking buffer, followed by 3 × 30 min washing in PBS at RT. Finally, sections were mounted with Fluorogold prolong antifade mounting medium (invitrogen).

### Single mRNA in situ hybridisation

mRNA in situ hybridisation was performed according to manufacturer’s instructions (ACDBio, RNAscope, Newark, CA); fluorescent in-situ hybridization for fixed frozen tissue, all reagents and buffers were provided by ACDBio [52]. Briefly, 40 μm tissue sections were mounted on superfrost plus glass slides- The slides were stored for at least 24 h at -80 °C and washed 2 × 5 min in 1 x PBS. The slides were baked for 30 min at 60 °C, followed by a post-fixation in 4% PFA for 15 min at 4 °C. The sections were dehydrated in a series of ascending ethanol concentrations (50% − 70% − 100%) for 5 min each, followed by a last time in 100% ethanol for 5 min. Sections were air dried and covered in 30% hydrogen peroxide for 10 min at RT, followed by 2x wash in PBS by moving the sections up and down 5x. Sections were incubated for 5 min in 1 x target retrieval solution at 100 °C, followed by a short wash step in dH20 and twice in 100% ethanol. Sections were covered in protease III solution and incubated for 30 min at 40 °C, followed by 2 × 2 min washing in dH20. The probes (C3, CD14, TNF, CCL2 and FOS) were added and incubated for 2 h at 40 °C, followed by a 2 × 2 min washing in wash buffer. Then, amplification 1 was added to the sections and incubated at 40 °C for 30 min followed by 2 × 2 min washing step with wash buffer. Next, amplification 2 was added to the sections and incubated at 40 °C for 30 min followed by 2 × 2 min washing step with wash buffer. As a final amplification step, amplification 3 was added and incubated for 15 min at 40 °C, followed by 2 × 2 min washing step with wash buffer. HRP corresponding to the channel was added to the sections and incubated for 15 min at 40 °C, followed by 2 × 2 min washing step with wash buffer. The diluted fluorophore (1:500 for all probes; in TSA buffer) was added to the sections and incubated for 30 min at 40 °C, followed by 2 × 2 min washing step with wash buffer. The remaining HRP was blocked by adding HRP-blocker to the sections and incubated for 15 min at 40 °C, followed by 2 × 2 min washing step with wash buffer. Sections were blocked using blocking buffer (3% BSA, 0.3% Triton X-100, PBS) for 1 h at RT, followed by primary antibody, diluted in blocking buffer, incubation overnight at 40 °C. The sections were washed 3 x with PBS-T (1% triton-x100) at RT for 20 min each, followed by secondary antibody incubation (diluted in blocking buffer) for 2 h at RT. The sections were washed 3 x with PBS-T at RT for 30 min each and mounted using Fluorogold prolong antifade mounting medium (Invitrogen).

### Image acquisition and analysis

Confocal images were acquired with a laser-scanning confocal microscope (LSM Leica DMi8 and Olympus Fluoview FV4000), with a 20x air objective (NA 0.75) and 40x oil objective (NA 1.3) in a 1024 × 1024-pixel resolution and a 12-bit (Leica) and 16-bit (Olympus) format. For the mRNA RNAscope imaging, a z-stack of 14–15 optical sections spanning 28–30 μm (step size of 2 μm), with a tilescan of 2 × 2 (40x objective) was made. For the microglia/pS6 imaging, a z-stack of 14–15 oP-TBIcal sections spanning 28–30 μm (step size of 2 μm), with a single tile (20x objective) was made. Imaging was done to obtain both the ipsilateral and contralateral side of the brain with 3 main regions (cortex, hippocampus and striatum). Imaging parameters were set to obtain signals from the stained antibody/probe while avoiding saturation. All fluorescent channels were acquired independently to avoid fluorescent cross-bleed.

Image analysis was performed using the ImageJ software. Images were background subtracted and smoothed. For the mRNA RNAscope analysis, images were contrasted to visualize all spots in the images, all spots per image were counted using the trackmate plugin (average spot size of 1 μm). Additionally, TNF/CCL2 positive microglia were assessed manually with the cell counter plugin, positivity of microglia was determined by having at least 2–3 spots of TNF/CCL2 inside the soma of the microglia. FOS positive neurons were assessed manually with the cell counter plugin, positive neurons were determined by having at least 5 spots of cFOS in NeuN positive cells. For the immunofluorescence analysis, pS6 intensity per microglia was assessed by thresholding the microglia and using the plugin ‘analyze particles’ with saving all region of interests (ROIs). The ROIs were overlaid in the pS6 channel and intensity was assessed per ROI. The Scholl analysis was performed on single microglia with a start radius of 0.622 um, a step size of 1.864 um and end radius of 45 um (size of insert used), the number of intersections per step size were automatically assessed. The total number of intersections per region and mouse were assessed and interquartile fractions were calculated, using the WT sham group as a reference.

### Statistical methods

Data analysis was performed using the Graphpad prism version 8 software or R software suits Version 4.3.1. Statistical analysis was performed using animals as a biological unit; whenever appropriate, single data points and averages per animal are depicted in the graphs. All datasets were tested for normality using the Shapiro-Wilks test. When comparing two groups, an unpaired t-test or Mann-Whitney test was used, based on normality. In-situ hybridisation and immunofluorescence data were analysed using a mixed effects model, comparing sham vs. P-TBI groups within each genotype, with a pre-specified pairwise testing with corrections (Holm-Šídák) comparing sham vs. ipsilateral and sham vs. contralateral in both genotypes. The Sholl data were analysed on the one hand by multiple t-test over distance from soma, and on the other by performing interquartile fractions based on the wildtype sham group followed by mixed effect model comparing the WT with the double knock-out within each region. qPCR and ELISA data (8 groups: 4 genotypes × 2 conditions) were analysed using a mixed-effects model including the factors genotype and condition and their interaction. Two families of planned contrasts were defined: (i) within-genotype comparisons of Sham versus P-TBI, and (ii) comparisons of the P-TBI response between genotypes, testing WT against each knockout line.* P*-values were adjusted for pre-specified pairwise testing with corrections (Holm-Šídák) within each family. Outliers were defined by the ROUT outlier test with a Q value of 1%. Significance was set at *p* < 0.05. Exact* p* values were reported where appropriate (with exception of the Scholl and hemodynamic analysis), descriptive statistical analysis with exact F values and* p* values for all datasets are reported in supplementary information descriptive statistics.

## Supplementary Information

Below is the link to the electronic supplementary material.

Supplementary Material 1

Supplementary Material 2

## Data Availability

All data supporting the findings of this study are available within the paper and its Supplementary Information.

## References

[1] Pape HC, Lefering R, Butcher N, Peitzman A, Leenen L, Marzi I, et al. The definition of polytrauma revisited: an international consensus process and proposal of the new Berlin definition. J Trauma Acute Care Surg. 2014;77:780–6. 10.1097/TA.0000000000000453. 25494433

[2] Teixeira PGR, Inaba K, Hadjizacharia P, Brown C, Salim A, Rhee P, et al. Preventable or potentially preventable mortality at a mature trauma center. J Trauma. 2007;63:1338–47. 10.1097/TA.0B013E31815078AE. 18212658

[3] Christie R, Cole E. Delirium after major trauma critical care and the association with recovery at 12 months. Trauma (London England). 2025;27:307–16. 10.1177/14604086251343660. PMC12490790 41050323

[4] Lee CC, Avalos AM, Ploegh HL. Accessory molecules for Toll-like receptors and their function. Nat Rev Immunol. 2012;12:168–79. 10.1038/NRI3151. PMC3677579 22301850

[5] Lupu L, Horst K, Greven J, Mert Ü, Ludviksen JAK, Pettersen K, et al. Simultaneous C5 and CD14 inhibition limits inflammation and organ dysfunction in pig polytrauma. Front Immunol. 2022;13:952267. 10.3389/FIMMU.2022.952267. PMC9433645 36059503

[6] Rani M, Sayyadioskoie SR, Galvan EM, Nicholson SE, Schwacha MG Trauma-induced lung injury is associated with infiltration of activated TLR expressing myeloid cells. Cytokine. 2021;141:155457. 10.1016/J.CYTO.2021.155457. 33581471

[7] Huber-Lang M, Lambris JD, Ward PA. Innate immune responses to trauma. Nat Immunol. 2018;19:327–41. 10.1038/S41590-018-0064-8. PMC6027646 29507356

[8] Burk AM, Martin M, Flierl MA, Rittirsch D, Helm M, Lampl L, et al. Early complementopathy after multiple injuries in humans. Shock. 2012;37:348–54. 10.1097/SHK.0B013E3182471795. PMC3306539 22258234

[9] Barrett CD, Hsu AT, Ellson CD, Miyazawa Y, Kong B, Greenwood YW. Blood clotting and traumatic injury with shock mediates complement-dependent neutrophil priming for extracellular ROS, ROS-dependent organ injury and coagulopathy. Clin Exp Immunol. 2018;194:103–17. 10.1111/CEI.13166. PMC6156817 30260475

[10] Barratt-Due A, Pischke SE, Nilsson PH, Espevik T, Mollnes TE. Dual inhibition of complement and Toll-like receptors as a novel approach to treat inflammatory diseases-C3 or C5 emerge together with CD14 as promising targets. J Leukoc Biol. 2017;101:193–204. 10.1189/JLB.3VMR0316-132R. PMC5166441 27581539

[11] Chung HY, Wickel J, Brunkhorst FM, Geis C Sepsis-associated encephalopathy: from Delirium to Dementia? J Clin Med. 2020;9:703. 10.3390/JCM9030703. PMC7141293 32150970

[12] Chen H, Mo L, Hu H, Ou Y, Luo J Risk factors of postoperative delirium after cardiac surgery: a meta-analysis. J Cardiothorac Surg. 2021;16:1496. 10.1186/S13019-021-01496-W. PMC8072735 33902644

[13] Flierl MA, Rittirsch D, Huber-Lang MS, Stahel PF Pathophysiology of septic encephalopathy–an unsolved puzzle. Crit Care. 2010;14:9035. 10.1186/CC9035. PMC2911737 20565858

[14] Mein N, von Stackelberg N, Wickel J, Geis C, Chung HY Low-dose PLX5622 treatment prevents neuroinflammatory and neurocognitive sequelae after sepsis. J Neuroinflammation. 2023;20:8. 10.1186/S12974-023-02975-8. PMC10691003 38041192

[15] Chung HY, Wickel J, Hahn N, Mein N, Schwarzbrunn M, Koch P, et al Microglia mediate neurocognitive deficits by eliminating C1q-tagged synapses in sepsis-associated encephalopathy. Sci Adv. 2023;9:7806. 10.1126/SCIADV.ABQ7806. PMC10219600 37235660

[16] 20 years TraumaRegister DGU(^®^). development, aims and structure. Injury. 2014;45(Suppl 3):S6–13. 10.1016/J.INJURY.2014.08.011. 25284237

[17] Toutonji A, Krieg C, Borucki DM, Mandava M, Guglietta S, Tomlinson S Mass cytometric analysis of the immune cell landscape after traumatic brain injury elucidates the role of complement and complement receptors in neurologic outcomes. Acta Neuropathol Commun. 2023;11:1583. 10.1186/S40478-023-01583-0. PMC10258994 37308987

[18] Alawieh A, Langley EF, Weber S, Adkins D, Tomlinson S. Identifying the role of complement in triggering neuroinflammation after traumatic brain injury. J Neurosci. 2018;38:2519–32. 10.1523/JNEUROSCI.2197-17.2018. PMC5858594 29437855

[19] Gustavsen A, Nymo S, Landsem A, Christiansen D, Ryan L, Husebye H, et al. Combined inhibition of complement and CD14 attenuates bacteria-induced inflammation in human whole blood more efficiently than antagonizing the toll-like receptor 4-MD2 complex. J Infect Dis. 2016;214:140–50. 10.1093/INFDIS/JIW100. PMC4907417 26977050

[20] Bjerkhaug AU, Granslo HN, Cavanagh JP, Høiland I, Ludviksen JK, Lau C, et al. Dual inhibition of complement C5 and CD14 attenuates inflammation in a cord blood model. Pediatr Res. 2023;94:512–9. 10.1038/S41390-023-02489-2. 36725909

[21] Meyuhas O, Ribosomal Protein S, Phosphorylation. Four decades of research. Int Rev Cell Mol Biol. 2015;320:41–73. 10.1016/BS.IRCMB.2015.07.006. 26614871

[22] Olde Heuvel F, Zhang J, Sun F, Krishnamurthy SS, Yartas G, Özkan B, et al Basophils activate splenic B cells and dendritic cells via IL-13 signaling in acute traumatic brain injury. J Neuroinflammation. 2025;22:3621. 10.1186/S12974-025-03621-1. PMC12709802 41408561

[23] Rehman R, Miller M, Krishnamurthy SS, Kjell J, Elsayed L, Hauck SM, et al Met/HGFR triggers detrimental reactive microglia in TBI. Cell Rep. 2022;41:111867. 10.1016/J.CELREP.2022.111867. 36577378

[24] Barratt-Due A, Pischke SE, Brekke OL, Thorgersen EB, Nielsen EW, Espevik T, et al. Bride and groom in systemic inflammation–the bells ring for complement and Toll in cooperation. Immunobiology. 2012;217:1047–56. 10.1016/J.IMBIO.2012.07.019. 22964230

[25] Ma Y, Jiang T, Zhu X, Xu Y, Wan K, Zhang T, et al Efferocytosis in dendritic cells: an overlooked immunoregulatory process. Front Immunol. 2024;15:1415573. 10.3389/FIMMU.2024.1415573. PMC11148234 38835772

[26] Marth T, Kelsall BL. Regulation of interleukin-12 by complement receptor 3 signaling. J Exp Med. 1997;185:1987–95. 10.1084/JEM.185.11.1987. PMC2196332 9166428

[27] Li Z, Zhang J, Halbgebauer S, Chandrasekar A, Rehman R, Ludolph A, et al Differential effect of ethanol intoxication on peripheral markers of cerebral injury in murine blunt traumatic brain injury. Burn Trauma. 2021;9:27. 10.1093/BURNST/TKAB027. PMC8484207 34604393

[28] Coelho LMG, Teixeira FJP, Koru-Sengul T, Manolovitz B, Taylor RR, Massad N, et al Predictive value of nervous cell injury biomarkers in moderate-to-severe traumatic brain injury: a network meta-analysis. Neurology. 2025;105:213997. 10.1212/WNL.0000000000213997. 40811753

[29] Chandrasekar A, Aksan B, Heuvel F, olde, Förstner P, Sinske D, Rehman R, et al. Neuroprotective effect of acute ethanol intoxication in TBI is associated to the hierarchical modulation of early transcriptional responses. Exp Neurol. 2018;302:34–45. 10.1016/j.expneurol.2017.12.017. 29306704

[30] Förstner P, Knöll B. Interference of neuronal activity-mediated gene expression through serum response factor deletion enhances mortality and hyperactivity after traumatic brain injury. FASEB J. 2020;34:3855–73. 10.1096/FJ.201902257RR. 31930559

[31] Walrath T, McMahan RH, Idrovo JP, Quillinan N, Kovacs EJ Cutaneous burn injury induces neuroinflammation and reactive astrocyte activation in the hippocampus of aged mice. Exp Gerontol. 2022;169:111975. 10.1016/J.EXGER.2022.111975. PMC10246470 36208823

[32] Rowe CJ, Nwaolu U, Martin L, Huang BJ, Mang J, Salinas D, et al Systemic inflammation following traumatic injury and its impact on neuroinflammatory gene expression in the rodent brain. J Neuroinflammation. 2024;21:3205. 10.1186/S12974-024-03205-5. PMC11360339 39198925

[33] Rowe CJ, Mang J, Huang B, Dommaraju K, Potter BK, Schobel SA, et al Systemic inflammation induced from remote extremity trauma is a critical driver of secondary brain injury. Mol Cell Neurosci. 2023;126:103878. 10.1016/J.MCN.2023.103878. 37451414

[34] Sankowski R, Mader S, Valdés-Ferrer SI Systemic inflammation and the brain: novel roles of genetic, molecular, and environmental cues as drivers of neurodegeneration. Front Cell Neurosci. 2015;9:28. 10.3389/FNCEL.2015.00028. PMC4313590 25698933

[35] Dalgard CL, Cole JT, Kean WS, Lucky JJ, Sukumar G, McMullen DC, et al The cytokine temporal profile in rat cortex after controlled cortical impact. Front Mol Neurosci. 2012;5:6. 10.3389/FNMOL.2012.00006. PMC3265961 22291617

[36] Taupin V, Toulmond S, Serrano A, Benavides J, Zavala F. Increase in IL-6, IL-1 and TNF levels in rat brain following traumatic lesion. Influence of pre- and post-traumatic treatment with Ro5 4864, a peripheral-type (p site) benzodiazepine ligand. J Neuroimmunol. 1993;42:177–85. 10.1016/0165-5728(93)90008-M. 8429103

[37] Chakraborty S, Winkelmann VE, Braumüller S, Palmer A, Schultze A, Klohs B, et al Role of the C5a-C5a receptor axis in the inflammatory responses of the lungs after experimental polytrauma and hemorrhagic shock. Sci Rep. 2021;11:79607. 10.1038/S41598-020-79607-1. PMC7835219 33495506

[38] Messerer DAC, Halbgebauer R, Nilsson B, Pavenstädt H, Radermacher P, Huber-Lang M. Immunopathophysiology of trauma-related acute kidney injury. Nat Rev Nephrol. 2021;17:91–111. 10.1038/S41581-020-00344-9. 32958893

[39] Ganter MT, Brohi K, Cohen MJ, Shaffer LA, Walsh MC, Stahl GL, et al. Role of the alternative pathway in the early complement activation following major trauma. Shock. 2007;28:29–34. 10.1097/SHK.0B013E3180342439. 17510601

[40] Paulus P, Rupprecht K, Baer P, Obermüller N, Penzkofer D, Reissig C, et al The early activation of toll-like receptor (TLR)-3 initiates kidney injury after ischemia and reperfusion. PLoS ONE. 2014;9:94366. 10.1371/JOURNAL.PONE.0094366. PMC3988056 24736450

[41] Sewell DL, Nacewicz B, Liu F, Macvilay S, Erdei A, Lambris JD, et al. Complement C3 and C5 play critical roles in traumatic brain cryoinjury: blocking effects on neutrophil extravasation by C5a receptor antagonist. J Neuroimmunol. 2004;155:55–63. 10.1016/j.jneuroim.2004.06.003. PMC4766842 15342196

[42] Janova H, Böttcher C, Holtman IR, Regen T, van Rossum D, Götz A, et al. CD14 is a key organizer of microglial responses to CNS infection and injury. Glia. 2016;64:635–49. 10.1002/GLIA.22955. 26683584

[43] Zhang H, Jin Q, Li J, Wang J, Li M, Yin Q, et al Astrocyte-derived complement C3 facilitated microglial phagocytosis of synapses in Staphylococcus aureus-associated neurocognitive deficits. PLoS Pathog. 2025;21:1013126. 10.1371/JOURNAL.PPAT.1013126. PMC12121917 40294039

[44] Zhang LY, Pan J, Mamtilahun M, Zhu Y, Wang L, Venkatesh A, et al. Microglia exacerbate white matter injury via complement C3/C3aR pathway after hypoperfusion. Theranostics. 2020;10:74–90. 10.7150/THNO.35841. PMC6929610 31903107

[45] Kim WG, Mohney RP, Wilson B, Jeohn GH, Liu B, Hong JS. Regional difference in susceptibility to lipopolysaccharide-induced neurotoxicity in the rat brain: role of microglia. J Neurosci. 2000;20:6309–16. 10.1523/JNEUROSCI.20-16-06309.2000. PMC6772569 10934283

[46] Jung H, Lee H, Kim D, Cheong E, Hyun YM, Yu JW, et al. Differential regional vulnerability of the brain to mild neuroinflammation induced by systemic LPS treatment in mice. J Inflamm Res. 2022;15:3053–63. 10.2147/JIR.S362006. PMC9140139 35645573

[47] Förstner P, Rehman R, Anastasiadou S, Haffner-Luntzer M, Sinske D, Ignatius A, et al. Neuroinflammation after traumatic brain injury is enhanced in activating transcription factor 3 mutant mice. J Neurotrauma. 2018;35:2317–29. 10.1089/NEU.2017.5593. 29463176

[48] Cursano S, Battaglia CR, Urrutia-Ruiz C, Grabrucker S, Schön M, Bockmann J, et al. A CRHR1 antagonist prevents synaptic loss and memory deficits in a trauma-induced delirium-like syndrome. Mol Psychiatry. 2021;26:3778–94. 10.1038/S41380-020-0659-Y. PMC8550963 32051550

[49] Moore KJ, Andersson LP, Ingalls RR, Monks BG, Li R, Arnaout MA, et al. Divergent response to LPS and bacteria in CD14-deficient murine macrophages. J Immunol. 2000;165:4272–80. 10.4049/JIMMUNOL.165.8.4272. 11035061

[50] Denk S, Weckbach S, Eisele P, Braun CK, Wiegner R, Ohmann JJ, et al. Role of hemorrhagic shock in experimental polytrauma. Shock. 2018;49:154–63. 10.1097/SHK.0000000000000925. 28614141

[51] Weckbach S, Hohmann C, Braumueller S, Denk S, Klohs B, Stahel PF, et al. Inflammatory and apoptotic alterations in serum and injured tissue after experimental polytrauma in mice: distinct early response compared with single trauma or double-hit injury. J Trauma Acute Care Surg. 2013;74:489–98. 10.1097/TA.0B013E31827D5F1B. 23354243

[52] Wang F, Flanagan J, Su N, Wang LC, Bui S, Nielson A, et al. RNAscope: A novel in situ RNA analysis platform for formalin-fixed, paraffin-embedded tissues. J Mol Diagnostics. 2012;14:22–9. 10.1016/j.jmoldx.2011.08.002. PMC3338343 22166544

